# Inorganic Materials as Supports for Covalent Enzyme Immobilization: Methods and Mechanisms

**DOI:** 10.3390/molecules190914139

**Published:** 2014-09-09

**Authors:** Paolo Zucca, Enrico Sanjust

**Affiliations:** 1 Consorzio UNO, Consortium University of Oristano, Oristano 09170, Italy; E-Mail: paolo.zucca@consorziouno.it; 2 Dipartimento di Scienze Biomediche, Università di Cagliari, Monserrato 09042, Italy; E-Mail: pzucca@unica.it

**Keywords:** enzymes, immobilization, stabilization, covalent binding, inorganic, silica, mesoporous, functionalization, grafting, activation

## Abstract

Several inorganic materials are potentially suitable for enzymatic covalent immobilization, by means of several different techniques. Such materials must meet stringent criteria to be suitable as solid matrices: complete insolubility in water, reasonable mechanical strength and chemical resistance under the operational conditions, the capability to form manageable particles with high surface area, reactivity towards derivatizing/functionalizing agents. Non-specific protein adsorption should be always considered when planning covalent immobilization on inorganic solids. A huge mass of experimental work has shown that silica, silicates, borosilicates and aluminosilicates, alumina, titania, and other oxides, are the materials of choice when attempting enzyme immobilizations on inorganic supports. More recently, some forms of elemental carbon, silicon, and certain metals have been also proposed for certain applications. With regard to the derivatization/functionalization techniques, the use of organosilanes through silanization is undoubtedly the most studied and the most applied, although inorganic bridge formation and acylation with selected acyl halides have been deeply studied. In the present article, the most common inorganic supports for covalent immobilization of the enzymes are reviewed, with particular focus on their advantages and disadvantages in terms of enzyme loadings, operational stability, undesired adsorption, and costs. Mechanisms and methods for covalent immobilization are also discussed, focusing on the most widespread activating approaches (such as glutaraldehyde, cyanogen bromide, divinylsulfone, carbodiimides, carbonyldiimidazole, sulfonyl chlorides, chlorocarbonates, *N*-hydroxysuccinimides).

## 1. Introduction

The term “enzyme immobilization” encompasses a wide range of laboratory and industrial processes aimed at retaining a fully active enzyme on a solid insoluble support [1,2,3,4,5,6,7]. In this light, the term “enzyme insolubilization” could also describe perfectly the aim of these techniques. There are several reasons to immobilize an enzyme: first of all, the efficient recovery of the catalyst after the reaction, and its immediate reuse for multiple catalytic cycles. Subsequently, the contamination of reaction products by the catalyst itself is also minimized (this is of crucial importance in pharmaceutical and food industries). Besides, immobilized enzymes usually feature enhanced specificity, selectivity [7,8], storage and operational stability [1,7,9] towards various denaturing agents (*i.e.*, extreme pH values, heat, organic solvents), and possibly prevent inhibition [8]. Lastly, only through immobilization, do multienzyme cascade processes become feasible [6,10].

All these features enable cost-effective uses of enzymes on industrial scale. Accordingly, immobilized enzymes find applications in several fields, such as biosensor production [2,11,12,13], bioproduct synthesis [3,6,14,15], bioethanol and biodiesel synthesis [13,16,17,18,19,20,21], pollutant removal [12,22,23,24], and biofuel cells [12,25,26].

Both organic (mainly polysaccharides, polyacrylic and polyvinylic materials) and inorganic supports (mainly silica- or other metal-oxide-based) have been described as efficient carriers for enzyme immobilization [3,6,27,28]. In particular, the latter are materials of choice in this field, and available with a wide range of porosities and costs. It is possible to chemically modify their surface enabling numerous immobilization techniques. Inorganic supports also present excellent thermal, mechanical and microbial resistance [3].

Several methods for enzyme immobilization have been proposed [2], including enzyme entrapment, cross-linking, and support binding. The latter can include physical bonding trough weak interactions (hydrogen bond, Van der Waals interactions), ion exchange, affinity interactions and covalent bonding [6]. Among all these methods, covalent immobilization generally ensures the highest strength of the bonding between support and enzyme, minimizing leakage issues. Moreover, covalent attachment does not usually interfere with reagents/products mass transfer, and allows the highest enhancement of operational stability (especially towards heat, pH, organic solvents, and also regarding the storage). These are crucial features in the feasibility of any industrial process.

**Figure 1:**
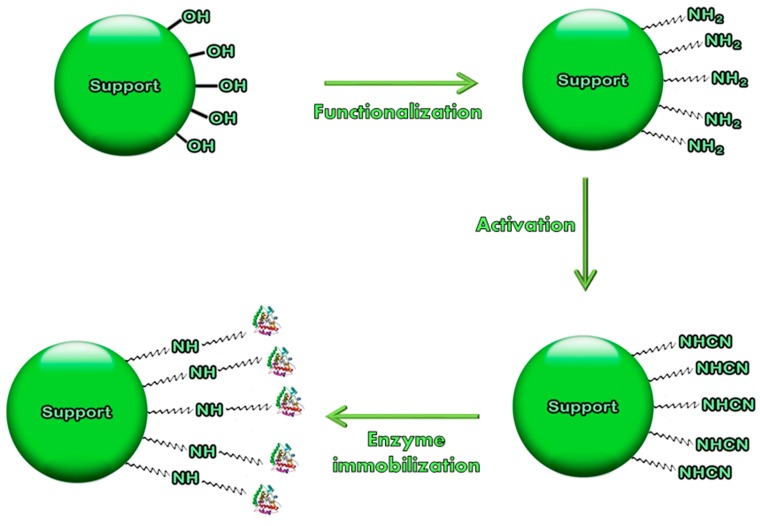
Scheme of functionalization and activation of inorganic supports during covalent immobilization (functionalization with –NH_2_ groups and activation with cyanogen bromide is reported as an example).

From this perspective, this review focuses on the most common methods for functionalization of inorganic supports. Several chemical functions can be inserted on the surface of the material (*i.e.*, –NH_2_, alcoholic –OH, –COOH, –SH) capable of covalently reacting with enzymes under proper conditions. Typically, following functionalization, activation of supports with specific activating agents (such as organic and inorganic halides, glutaraldehyde, carbodiimides, various bifunctional agents) is necessary, to achieve enzyme immobilization. Conceptually, functionalization or derivatization is the procedure by which a new chemical function is introduced onto a support (enzyme carrier). Activation means that the newly introduced chemical function is made reactive towards the enzyme. Sometimes, functionalization and activation coincide. The scheme for covalent immobilization on inorganic supports is summarized in Figure 1. Many methods of activation have been also described, that help minimizing the typical drawbacks of covalent immobilization (loss of activity, modification of 3-D protein structure, use of toxic functionalizing/activating agents) [2], and are reviewed in this paper. Furthermore, the operational conditions of these reactions are examined in depth, in the perspective of rendering the whole processes industrially and commercially viable.

The choice of the proper support greatly affects the whole feasibility of industrial applications for an immobilized enzyme. The chosen support should fulfill some crucial requirements to be suitable for covalent immobilization:

- The support should be relatively inexpensive and environmentally harmless, minimizing the economic impact of the process.
- The support should be able to load a significant amount of enzyme per unit of weight. Accordingly, porosity could be a beneficial feature, but the diameter of the pores has to remain within a proper range (wider than the average protein diameter), since smaller pores merely exclude the protein, and too large ones will cause a significant drop in the surface area. In both cases the loading capacity is adversely affected [3,9,29]. High surface area and proper particle size should be also considered [30,31].
- Hydrophobicity of the surface should be usually minimized, since it favors undesired protein adsorption and denaturation [24,31]. Contrary behavior has been described only for well-known hydrophobic enzymes, such as lipases [8,32]. Generally speaking, the support should present the optimal micro-environment to enhance the catalytic features of the immobilized enzymes [8].
- Functionalization and activation require reactive chemical functions on the surface of the support. These groups should present minimal steric hindrance (especially for multi-point attachment [7]) and high superficial density.
- After immobilization, the support should be however totally inert under the enzymatic operational conditions, not interfering with the desired reaction.
- Interferences by unspecific protein/support interactions (*i.e.*, adsorption, ion exchange) should be minimized, except in the case of specifically desired multifunctional immobilization [33].
- Microbial resistance is mandatory for a commercially viable enzyme.
- Thermal and mechanical resistance are also important, enabling immobilized enzyme to be used under different operational conditions [29]. Particularly, resistance to abrasion (for batch reactors) and flow pressure (for continuous reactors) should be taken into due account [30,34].
- Chemical durability should be also considered. For instance, pH values far from neutrality could significantly affect the stability of inorganic structures [35].

Immobilization supports are commonly divided into two main classes: organic and inorganic [6]. The first group includes mainly polysaccharides (such as modified celluloses, dextran, chitosan and agarose [36,37]), vinylic and acrylic polymers (such as polyacrylamide and poly(vinyl alcohol) [38,39]). Polyamides, such as nylon, also find applications in this field [40,41].

## 2. Inorganic Supports 

Among inorganic supports, several silica-based and other oxide-based materials are accounted. These are widely considered the materials of choice for enzyme insolubilization since they are endowed with all the features mentioned above. For instance, the thermal and mechanical resistance of inorganic supports are generally higher [30]. Whereas microbial resistance is typically complete, as inorganic materials are not substrates at all for any kind of bacterial/fungal growth [30]. Furthermore, two main features distinguishing inorganic supports are rigidity and porosity. Organic materials can also be obtained with strictly controlled porosity, but they are usually very sensitive to pressure or pH, or in many cases to both. On the contrary, the typical stiffness of the inorganic supports ensures the invariance of pore diameter/pore volume, which guarantees constant volume and shape to the support itself. Inorganic carriers showing various pore diameters are commercially available. Nonetheless, the most promising materials are mesoporous supports, having pores ranging between 2 and 50 nm in size, and surface areas starting from 300–500 m^2^·g^−1^ and up to 1500 m^2^·g^−1^[3,4,42]. This order of magnitude of diameters (ranging approximately around the average diameters of proteins) is supposed to enable the highest protein loadings during immobilization [9,43].

### 2.1. Silica-Based Supports

#### 2.1.1. General

A detailed discussion about the properties of silica [44,45] is beyond the scope of this review, and therefore only some reminders of its outstanding features, related to enzyme immobilization techniques, are presented here. The general properties and the chemistry of silica surfaces have been recently covered in exhaustive reviews [46,47]. Contrarily to carbon dioxide, which is a typical example of a molecular dioxide, silicon dioxide usually exists as a 3-D polymer, whose units are regular SiO_4_ tetrahedra with shared their vertices. Therefore, the whole structure is an infinite lattice where siloxane bridges Si–O–Si are the only bonds found. The SiO_4_ tetrahedra are quite rigid entities; by contrast, the Si–O–Si angles are highly flexible (difference from C–O–C ones), which explains the hundreds of known polymorphs of silica, ranging from highly ordered crystalline forms (such as quartz) to totally random structures, such as vitreous (glassy) materials, passing through non-periodic porous systems to microporous crystalline and mesoporous amorphous solids.

SiO_2_ exists in two main crystalline forms, quartz and cristobalite, being the so-called tridymite merely considered a variant of quartz. Moreover, the compound has a noticeable tendency to exist as an amorphous material. Under exceptionally high temperature and pressure, a quite different SiO_2_ form is obtained, which is also known as a natural mineral: stishovite [48,49]. This is an ultra-dense metastable form where each silicon atom is hosted within an octahedral cluster of six oxygen atoms, such as it is observed in the rutile form of titania (titanium dioxide, TiO_2_, *vide infra*). Stishovite is extremely compact and therefore unreactive, to the point of being almost unaffected by HF. Conversely, when SiO_4_ tetrahedra share edges, such as in fibrous silica [50,51], four-membered (Si_2_O_2_) rings arise. These are also formed under fracture of bulk silica pieces, either amorphous or crystalline, and are responsible for a noticeable chemical reactivity.

#### 2.1.2. Silica Surface Chemistry

Silica surfaces can arise from dehydration of hydrated silica preparations (“silicic acids”) or from grinding bulkier silica pieces. In the former case, water elimination takes place as the temperature rises and condensation of silanol functions Si–OH produces siloxane bridges Si–O–Si [52]. At about 1500 °K, virtually all silanol groups are eliminated, and a sintering process takes place. The silica network, to a certain extent, rearranges and compacts, rendering the rehydration process extremely slow [53]. On the other hand, any fracture in a silica piece, regardless to its amorphous or crystalline structure, produces new surfaces, showing the effect of a reconstruction process that leads to strained rings and/or thermodynamically and kinetically unstable functions such as silanones >Si=O [54]. All these structures, even in the presence of minimal water concentrations, are subjected to hydration processes leading to the formation of silanol groups. Ultimately, the commonly observed silica surfaces show two main chemical functions: silanols (Si–OH), and siloxanes (Si–O–Si). Both are influenced in their properties by the electronic structure of silicon. Its empty 3*d* orbitals could host electronic density from the bound oxygen atoms. As a consequence, the Si–O bond is shorter than expected, and a noticeable *p*π–*d*π double bond character could be envisaged [55]; in other words, the expected *sp*^3^ hybridization of the oxygen atoms could shift to a certain extent toward *sp*^2^ one, explaining the wide range of the observed Si–O–Si angles. Therefore, the oxygen in a siloxane moiety is a quite poor Lewis base [56]. Also in silanols the Si–O bond is shorter than expected, for the same reasons as above. The oxygens are therefore less electron-rich, although still capable of protonation or taking part in hydrogen bonding as a proton acceptor. Anyway silanols are significantly more acidic than their alcohol counterparts, thus rendering silica surfaces negatively charged within a wide range of pH values, and moreover show a remarkable tendency to act as donors in hydrogen bonding [57]. Hydrated silica behaves as an acidic oxide when exposed to basic solutions, and therefore it shows a tendency to go into solution: OH^−^ ions gradually break siloxane bridges and a solution containing alkaline silicates is obtained [58]. The reaction is relatively fast for amorphous, powdered silica, but extremely slow for quartz.

**Figure 2:**
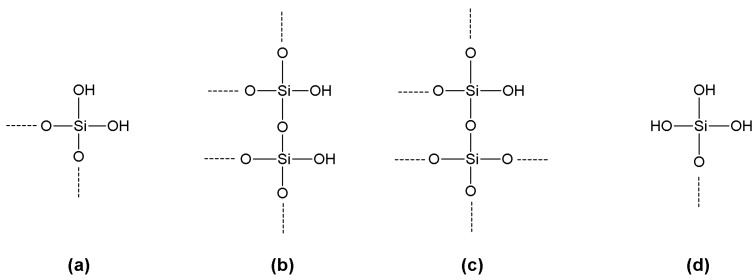
Several types of silanol functions can be found on the surfaces of silica-based materials: geminal silanols (**a**), vicinal silanols (**b**), isolated silanols (**c**). Silanetriols (**d**) have never been found on silica surfaces.

In principle, a regular layer of silanol groups should cover crystalline silica; in fact, owing to defects in the crystalline lattice and other irregularities of the surface, where the structure is amorphous, regions of relatively high silanol concentrations exist together with others where siloxane motifs sharply prevail. These latter are responsible for the observed hydrophobic character of some silica preparations [46]. Various types of silanol functions could be classified as the reactive groups at the surfaces of silica-based materials: geminal silanols (silanediols), vicinal silanols, isolated silanols (Figure 2). Silanetriols –Si(OH)_3_ have never been found on silica surfaces [59]. With regard to inter-silanol hydrogen bonding, reciprocal bonding between the two hydroxyls of geminal silanediols does not take place, owing to their *anti* orientation. Despite of the favorable distance, also in the case of vicinal silanols reciprocal hydrogen bonding is not always observed.

The coexistence of both hydrophobic and hydrophilic sites on silica surface, and the particular properties of the latter (weakly acidic, and with high tendency to take part in hydrogen bonding) explain the complex adsorptive properties of many silica-based materials. This is quite relevant in the context of this review, since adsorption is usually observed as an undesired phenomenon when covalently immobilizing enzymes onto silica surfaces [60].

#### 2.1.3. Siliceous Porous Materials

A noticeable aptitude for forming porous structures is an outstanding feature of silica-based materials [4]. Pore diameters can be varied within a very wide range, from micropores (<2 nm) to mesopores (<50 nm) to macropores (>50 nm) [61]. Zeolites are typical examples of siliceous microporous supports [62], but they are nearly irrelevant with respect to enzyme immobilization owing to their too narrow pores [15,27,30]. On the other hand, too large pores often involve a relatively low surface area, preventing high enzyme loadings [63], so mesoporous silica-based materials have become very popular materials for immobilizing proteins [30]. In principle, mesoporous supports could be ordered, with a periodic arrangement of regular cavities and walls, or disordered, with a casual distribution and orientation of cavities within the structure. As a point of fact, up to date, ordered structures greatly facilitate physicochemical and structural characterization of the matrices, whereas a real advantage with respect to enzyme loadings is still waiting for a conclusive assessment [4]. Anyway, mesoporous supports not only show a very high specific surface (up to 600 m^2^/g), but the enzyme molecules are hosted within pores [27], and are therefore protected against physical and mechanical damage.

A defined procedure to prepare mesoporous silica has been known since 1967 [64], but it received almost no attention. In 1990, an independent synthesis of a mesoporous silica was reported [65], and the obtained materials were further characterized [66], being named Folded Sheet Materials (FSMs). Later, new mesoporous silica-based materials (Mobil Compositions of Materials, also known as Mobil Crystalline Materials, MCMs), showing different structures depending on the preparation parameters [67] were developed at Mobil [68,69,70], followed by others discovered at the University of California, Santa Barbara, and named after the institution as Santa Barbara Amorphous materials (SBAs) [71,72]. Such materials are generally prepared by controlled hydrolysis of tetraethyl orthosilicate (and sometimes, instead, of Na_2_SiO_3_ [73]) in the presence of significant concentrations of suitable surfactants in water/organic solvent solutions, where the micellar nature of the surfactants drives the nascent silica network to form highly ordered, mesoporous structures. Roasting in the presence of excess air eliminates the templating organic surfactants. Spongy and totally inorganic structures survive, saving the original 3-D arrangement of the starting hydrogels. Alkaline hydrolysis (with aqueous ammonia) in the presence of cetyltrimethyl ammonium bromide as the surfactant leads to the commonest member of the MCM family, namely MCM-41. The use of the non-ionic surfactant P-123 (Pluronic^®^) and aqueous HCl leads to the most popular SBA material,* i.e.*, SBA-15. An impressive number of articles deals with the growing field of ordered, mesoporous, silica-based particles; the reader is referred to a recent review for synthesis, properties, and applications of such materials [74]. Both MCM-41 and SBA-15 could easily accommodate large molecules such as proteins and enzymes, which explains their huge popularity. Other silica-based mesoporous materials have been described, such as Michigan State University (MSU)-1 [75] and Mesostructured Cellular Foam (MCF) [76], although they have drawn no attention as tools for protein immobilization, perhaps owing to their disordered structure.

Generally speaking, not only advantages could be found when working with mesoporous, silica-based materials as supports for enzyme immobilization. In fact, they show very high enzyme loadings, and the enzyme molecules, hosted within channels, are protected from microbiological attack as well as from physical/mechanical damage. On the other hand, highly porous supports are unavoidably fragile and prone to grinding when subjected to strong pressures. Moreover, diffusion issues affect the molecular traffic (of substrates and products of the enzymic process) between inside and outside the channels. In certain cases, a given enzyme could be easily accommodated within a channel, but its catalytic activity will be low owing to excessive crowding of enzyme molecules onto the (convex) inner surface of the channel, affecting its catalytic efficiency; in other cases, one enzyme molecule could simply obstruct the pore, making the corresponding channel space—and worse the other enzyme molecules therein—useless [27]. However, in some specific cases diffusional issues may also turn into positive outcomes [8].

#### 2.1.4. Controlled Pore Glass (CPG)

It is common knowledge that, upon suitable thermal treatment, alkaline borosilicate glasses (where sodium is the usual alkaline cation), constituted by a proper balance among Na_2_O, B_2_O_3_, and SiO_2_ form two intimately and mutually compenetrating phases. These phases could be easily separated as the one richer in sodium and boron is soluble in water, whereas the other one (96% SiO_2_ and 4% B_2_O_3_) is insoluble and remains as a spongy vitreous hard matter after soaking with an aqueous dilute mineral acid (also the “insoluble” phase, could be slowly solubilized when soaked with alkaline solutions) [77]. Depending on the particular thermal treatment, a wide range of pore diameters could be obtained, usually from about 30 to more than 1000 Å. These could be enlarged to a certain extent by a controlled alkaline treatment to dissolve a loosely bound silica fraction within the pores [78]. The pores give access to syndetic channels that would host different molecules such as enzymes. The silanol groups coating the channel walls show a chemistry quite paralleling that of the silica-based materials described above. However, the presence of a low percentage of B_2_O_3_ in the glass mass has interesting consequences for the overall surface properties. A defined tendency of boron to concentrate along the surfaces has been observed, leading to a Lewis-type acidic centers that cooperate with the weakly Brönsted-type acidic centers (silanols) to strongly bind basic molecules. CPGs share with most siliceous supports a tendency to pass into solution when exposed to sharply alkaline conditions. Different CPG preparations are commercially available and, although costly, are highly popular as supports for enzyme immobilization [79,80].

#### 2.1.5. Fumed Silica

The so-called fumed silica represents a completely different approach to the preparation of inorganic supports with high specific surface, featured by high protein loadings. This material is obtained by combustion at very high temperatures of SiCl_4_, injected in a flame. Out of the flame, the cooling of the obtained fused silica droplets is so rapid that a vitreous material is obtained, where the primary droplets aggregate to form branched chains, that in turn can aggregate to form new superstructures. The fast cooling along the preparation of the product prevents any structural organization and/or reconstruction, so the surface is highly reactive and fumed silica is decidedly hygroscopic. Fumed silica preparations have very high specific surface areas, owing to the very small particles. The facile hydration process in the presence of water leads to a massive presence of silanols, which explains their absorptive properties towards proteins [81,82]. Hydrated fumed silica is decidedly more hydrophilic than other silica-based materials. Accordingly, hydrogen bonds and electrostatic interactions are the major types of bonding responsible for the stubborn adsorptive properties. In principle, the same reactivity could permit chemical functionalization in the view of covalent enzyme immobilization, provided that the unwanted, non-specific adsorption phenomena could be kept to a minimum by the particle coating formed with the derivatization procedure.

#### 2.1.6. Silica-based Nanoparticles

Although fumed silica could be well defined as a nanosized material, silica nanoparticles are usually produced also at laboratory scale by taking advantages from the well-known sol-gel methods [83,84,85,86,87]. Nanoparticles are characterized by their very high surface/volume ratios, so their properties are often quite different from the corresponding bulk molecules. Consequently, they have drawn much interest in many research and technology fields. Silica nanoparticles tend to aggregate when dried from an aqueous suspension—as observed for hydrous fumed silica—thus partially wasting the advantages of a very high surface area; conversely, their recovery from aqueous suspensions could be very tedious, preventing several immobilization applications [88]. A fine tuning of particle size, surface properties, and porosity could be achieved when a judicious choice of starting materials and preparation procedures is made, opening the way for a huge number of applications, ranging from catalytic systems to sensing and drug-delivery devices [89,90]. In fact, enzyme immobilization on silica nanoparticles is mainly devoted to, but not restricted to, selective biosensing of a wide range of analytes [91,92,93,94,95].

### 2.2. Ceramics

The term “ceramics” broadly indicates solid, insoluble, inorganic, non-metallic materials, usually based on metal oxides and/or mixed metallic and non-metallic oxides, obtained by “cooking” plastic and moldable (they contain water) starting mixtures. The original meaning of the word refers to various kinds of substances, documented for thousands of years, and known as terracotta, faience, porcelain, china, and bone china. The cooking process, with temperatures varying within a wide range, is not merely a dehydration process, but involves more or less deep changes in the chemical nature of the starting material, and is generally speaking a “roasting” (strong heating in the presence of excess oxygen – commonly air) to destroy all the possible organic matter, rather than “calcining” (strong heating under lack of oxygen conditions). Siliceous glasses are usually regarded as a kind of ceramics, although partial or total crystalline nature is among the features of ceramics from a more rigorous point of view. Following both a strict and traditional definition, the term “ceramics” should be reserved for those products, based on silicoaluminates such as kaolinite [96], montmorillonite [97], and others [98]. More recently, metal or semimetal oxides such as Al_2_O_3_, TiO_2_, ZrO_2_, SnO_2_ and others have also been referred to as “ceramics”, regardless to their thermal history [99,100,101,102,103].

Ceramics’ porosity varies widely in function of the starting material, time and temperature of the cooking process: so strong heating for prolonged times generally leads to a vitrification process with concomitant sintering, which greatly reduces the porosity. Conversely, products obtained at comparatively low temperatures, such as terracotta, are highly porous, but soft and easily crumbled. By a judicious choice of starting mixtures and cooking conditions, many porous ceramic materials, potentially useful for enzyme immobilization, could be obtained [104,105]. Also surface modification of the particles and/or of the channels of the porous materials has been described [106,107,108,109,110].

### 2.3. Titania and Zirconia

Titania is titanium dioxide TiO_2_. Titanium is bulkier than silicon, which explains its usual coordination number (6) compared with that of silicon (4). Titania exists in three main crystalline modifications: rutile (stable), anatase and brookite (metastable forms). Owing to the higher metallic character of titanium, titania is more basic and less acidic than silica, and resists exposure to alkaline solutions. In contrast to silica, titania tends to exist as a crystalline material, also when it is obtained by a sol-gel method [111]. However, amorphous, hydrous titania nanoparticles have been easily obtained and characterized [112]. Generally speaking, anatase is the sole product obtained when operating below 80 °C, whereas rutile tends to become the main product as the temperature rises, so that at ≈800 °C only rutile is formed. Pure anatase particles could be obtained by controlled titanium alkoxide hydrolysis at 90–100 °C [113]. The surface of titania particles is characterized by the presence of ≡Ti–OH groups. These could be gradually eliminated upon strong heating, even if a small number still survives even at 700 °C in vacuum. Re-hydroxylation of the surface by soaking in water takes place, at least in part, but repeated cycles of hydroxylation/dehydroxylation lead to lowering of ≡Ti–OH density, owing to stabilization of Ti–O–Ti bridges by surface restructuring [114]. In general, titania surfaces are chemically reactive and could undergo covalent modification as the first step for enzyme immobilization (*vide infra*). Accordingly, many studies have been published on the use of titania (nano)particles as supports for enzyme (covalent) immobilization [111,115,116]. Under certain conditions, titania could be partially and reversibly reduced to Ti_2_O_3_ in a redox reaction which could explain the high photocatalytic activity of titania-based materials [117].

Zirconium shows many analogies with titanium, and the same is true for the corresponding dioxide (ZrO_2_, zirconia). However, zirconium is decidedly a metal, so zirconia lacks any acidic character at all, and is correctly considered a slightly basic, insoluble metal oxide. Consequently, it resists attempts of stable covalent modification, so its importance in enzyme covalent immobilization is marginal [118]. Zirconia has instead interesting properties as a support for enzyme adsorption [119].

### 2.4. Alumina

Alumina is aluminum oxide Al_2_O_3_, widely existing in Nature as corundum, and as the component of many aluminum ores, usually in its hydrous forms. Many alumina preparations have been well-known for many decades due to their useful catalytic properties for industrially relevant gas-phase reactions, so their physicochemical structural and surface properties have been deeply studied [120,121,122]. Alumina is usually obtained by strong heating of the hydroxide Al(OH)_3_, which exists in several different crystalline forms and—transiently—as an hydrogel when precipitated from aqueous aluminum salts upon base treatment; depending on the particular hydroxide used as the starting material, different crystalline forms of alumina could be prepared. All these forms, usually characterized by their sandy appearance, high porosity and high specific surface, change into α-alumina (corundum), which is a white, floury product, showing almost no porosity and a very low specific surface, when heated at >1000 °C. α-Alumina is quite inert, and devoid of any interest in the field of protein immobilization, and as expected shows no any catalytic activity. “Sandy” alumina, obtained by moderate (400 ÷ 800 °C) calcination, exists in various crystalline modifications (being η and γ the best known ones), which could represent interesting alternatives to silica-based supports. Alumina surfaces obtained by dehydration of hydroxides are coordinatively unsaturated, which explains the “acidity” of these preparations. This feature is important for both catalytic and adsorptive properties. However, alumina is only seldom found as a support for enzyme immobilization procedures [123,124,125]. Perhaps, the tendency of sandy alumina to crumbling has discouraged a systematic exploration of its potential utility in enzyme immobilization.

### 2.5. Magnetic Supports

Magnetic supports show the obvious feature of being ferromagnetic, which allows recovery of the immobilized enzyme preparation from a reactor by simply applying a magnetic field to the slurry, without the need of tedious decantation, centrifugation, or filtration procedures. Metallic iron is not suitable as a support—unless coated with inert and impermeable materials—owing to its facile oxidation to ferric hydrous oxide upon exposure to water in the presence of air. Obviously, stainless steel has a totally different behavior. Magnetite (Fe_3_O_4_) is a well-known iron mineral, forming the most valuable iron ores; it is a hard, blackish, insoluble, poorly reactive solid, but can be directly prepared in laboratory in the form of nanoparticles [126,127]. Magnetite could otherwise be prepared as particles of suitable size, so they could be directly used for covalent enzyme immobilization, or—more frequently—incorporated within particles such as silica-based materials, that are the true reacting support for enzyme immobilization. Magnetite is not the only metal oxide potentially suitable as magnetic material for immobilization experiments; other supports have been described and used after direct functionalization or also after coating with the “true” immobilization support. Among these materials, nickel and cobalt ferrites show interesting magnetization, hardness, and chemical inertness properties [128,129,130,131].

### 2.6. Other Inorganic Supports

Elemental silicon, which easily undergoes surface oxidation when exposed to air, is an obvious alternative to silica for enzymatic electrosensing [132]. However, silicon oxidation goes further, leading to formation of hydrous silica and, on the whole, to electrode deterioration, so the technique is not very popular.

Other solid inorganic materials are occasionally used as supports for covalent immobilization of enzymes; among these, tin dioxide SnO_2_ can be cited. This is found in Nature as the mineral cassiterite, which is industrially relevant as the most abundant tin ore. SnO_2_ is a heavy white powder, insoluble in water, but soluble in strong alkaline solutions; it can be precipitated from aqueous media in the form of hydrous (nano)particles, useful to immobilize enzymes [133].

Metallic gold, although expensive, as a noble metal it is exceptionally stable towards nearly all reagents, and is used in immobilization techniques. The very high electrical conductivity of gold makes the metal the perfect support for the construction of electro-enzymatic sensors [134,135]. Moreover, gold could be easily molded into extremely thin foils, or in colloidal suspensions of nanoparticles. Fortunately, although chemically inert, gold has a special affinity for mercaptans, so bifunctional organic reagents, bearing at least one thiol function, could form a chemical “bridge” between the metal (nano)particle and the enzyme to be immobilized.

In principle, other metals could be used for enzyme immobilization, provided that their surface is reactive enough for covalent modification/activation. Among these, titanium could be oxidized at its surface, in a manner that produces a monomolecular titania layer. The latter is the true support for the immobilization reactions [136].

With regard to polyphosphazenes, these polymers should be regarded as inorganic/organic species. Although described as supports for enzyme immobilization [137], their organic moieties are inherently part of the whole molecule rather than tethers added along functionalization/activation procedures, so they are beyond the scope of the present review.

## 3. Advantages and Drawbacks of Covalent Enzyme Immobilization

Several approaches for enzyme immobilization have been proposed, as summarized in Table 1.

(i) *Encapsulation* and *entrapment* do not involve chemical bonds between the support and the protein, which is in fact simply included in the 3D network of the support, making impossible its diffusion away from the carrier. Accordingly, minimal modification of the native structure is involved, but leakage of enzyme is often observed [2]. Besides, mass transfer issues can often occur, involving both substrates and products.
(ii) *Adsorption* and *electrostatic* *interaction* are often overlapping phenomena due to non-specific weak interactions, still not completely clarified [27,138]. However, the simplicity of this approach and the low modification of protein surfaces are responsible for the wide diffusion of such techniques [46]. Unfortunately, the non-specificity of the interactions could lead to unexpected leakage resulting from changes in several operational parameters (pH, temperature, and ionic strength particularly), thus suggesting the application of physically adsorbed enzymes mainly in hydrophobic environments [30].
(iii) *Cross-linked* *enzymes* (CLEs) (such as cross-linked enzyme crystals CLECs, or aggregates CLEAs) involve the formation of covalent bonds among protein molecules using bifunctional reagents [6,9], often avoiding the use of any carrier. Glutaraldehyde and *bis*(imidoesters) are the most used bifunctional cross-linking agents. The covalent nature of the interaction is reflected in the minimal leakage and boosted operational stability of the enzymes (also under harsh conditions) [30], whereas the negative side is the possible chemical modification of the protein surface. Substrate/product diffusion rates can be also affected, and use of toxic reagents under complicated reaction conditions are often necessary [30].
(iv) *Affinity interaction* between ligand-grafted carrier and protein can represent a valid alternative [139,140], since it could allow high-strength bonding (and so minimal leakage), without affecting a protein’s native structure [141]. Unfortunately, this approach requires the presence of specific chemical functions on the protein and a different carrier grafting for each protein, often rendering its broad diffusion for industrial enzymes uneconomical.
(v) *Covalent* *attachment* tops the other approaches concerning the strength of the interactions, typically minimizing protein leakage. Several aminoacid side chains can form covalent bonds with activated inorganic supports. Particularly, the widespread lysine ε-NH_2_. Massive structural modifications of the immobilized proteins are accordingly likely to occur. Even when this is excluded, the simple bad orientation of the active site could affect the proper interaction between enzymes and substrates [24]. All these phenomena could thus affect catalytic activity.

**Table 1:** Advantages and disadvantages of the most common methods of enzyme immobilization.

Method of Immobilization	Advantages	Disadvantages
**Encapsulation/entrapment**	No chemical modification of the enzyme Enzyme should retain catalytic activity under the conditions of polymerization/transition of the support	Enzyme leakage Mass transfer issues
**Enzyme cross-linking**	No support is needed Stabilization of the enzyme Minimization of catalyst leakage	Possible massive chemical modification of the enzyme Complicated experimental processes Mass transfer issues
**Adsorption**	No chemical modification of the enzyme Easy and cheap to be performed	Enzyme leakage Low specificity of the reaction (*i.e.*, adsorption and ion-exchange could overlap)
**Electrostatic interaction**	No chemical modification of the enzyme Easy to be performed	Enzyme leakage Low specificity of the reaction (*i.e.*, ion-exchange and adsorption could overlap)
**Affinity**	High specificity of the reaction	The presence of specific groups on the enzyme is mandatory Usually expensive and complicated to be designed
**Covalent binding**	Strength of the binding Minimization of catalyst leakage Stabilization of the enzyme	Possibility sterical modifications of the enzyme Decrease of enzymatic activity is possible Chemical modifications of the support are necessary Usually irreversible attachment, preventing support reuse

On the other hand, stiffening of the 3D protein structure usually enhances its apparent stability towards several operational parameters (organic solvents, pH, ionic strength, temperature) [9], especially in the case of multipoint attachment [7,8]. Distortion of polypeptide chains can also be reflected in positive modulation of specificity, selectivity and decrease of inhibition [7,8]. Besides, a covalent approach could more easily allow the co-immobilization on the same support of multiple enzymes or enzyme/cofactor combinations for tandem reactions or cofactor recycling systems [30]. Covalent interactions with the support can also result in stabilization of multimeric enzymes, avoiding subunit dissociation [142]. Covalent immobilization, however, requires usually both functionalization and activation of the support. All these steps need to be properly designed to avoid all the above-mentioned drawbacks and to afford the highest increase in protein stability and activity. In fact, badly designed immobilization process can also result in the opposite effect (*i.e.*, decrease in stability) [33,143]. Covalent attachment of enzymes is usually irreversible, therefore preventing support reuse when the immobilized enzyme has lost its activity. However, in certain cases protein release under mild conditions is possible, thus achieving support recovery [§ (Sections) 4.3, 4.4, 5.1, 5.3].

## 4. Functionalization of Inorganic Supports

Typically, a generic support for protein immobilization should be chemically inert, otherwise, it could react with water (or buffers), and/or with substrates/products arising from the enzyme-catalyzed reactions. However, an ideal support should be reactive enough to be chemically modified with a view of achieving enzyme immobilization [144]. Restricting inspection to inorganic supports, they usually show a more or less hydrated surface, consisting of –OH groups covalently bound to the atoms, typical for the particular support examined. There is a great difference between organic polyhydric supports, such as polysaccharides and poly(vinyl alcohol), that display primary and/or secondary alcoholic functions, and all inorganic supports, whose –OH groups behave quite differently from alcohols.

Generally speaking, the semi-metallic or decidedly metallic character of the elements involved in the formation of the corresponding (hydrous) oxides affects all these supports. Moreover those elements do not obey the octet rule, having empty *d* orbitals hosting electronic density from nucleophiles such as water. As a consequence, bonds such as Si–O–C, Sn–O–C, Ti–O–C, Al–O–C and so on, very easily undergo S_N_2-type reactions at the heteroatoms, leading to hydrolysis under the conditions of subsequent enzyme immobilization. Therefore, these entities are more correctly classified as alkoxides rather than ethers (a crucial difference with respect to the organic supports mentioned above). As such, those alkoxide bonds are generally not suitable—although with some noticeable exceptions, *vide infra*—for stable derivatization of supports and immobilization of enzymes.

Silanol functions tend to behave as slightly acidic ones, for the reasons outlined in § 2.1. Ti–OH, Al–OH, and Sn–OH are less acidic and more basic (in the Brønsted sense), but on the whole their reactivity is not too different from that of silanols. As a consequence, some inorganic supports based on semi-metal and metal oxides are more or less easily dissolved by strongly acidic or alkaline solutions, which is another important limitation when seeking to properly functionalize these supports.

### 4.1. Silanization: General

Organosilanes are the reagents of choice for stable and reliable functionalization of most organic supports; in principle, three main approaches could be adopted to achieve support functionalization [42]:

(i) *Grafting*: the plain support is treated under suitable conditions with a chosen organosilane, forming some sort of covalently bound coating. This coating is formed by the organic functions of the starting silane;
(ii) *Co-condensation*: support particles such those described in § 2, are synthesized by means of sol-gel procedures, starting from a proper mixture of tetraethyl (or tetramethyl) orthosilicate and the chosen trialkoxyorganosilane. Tetraalkyl orthosilicates can be replaced by other alkoxides such as tetraethoxytitanium or so on. The growing particles incorporate the added organosilane and a very regular distribution of the organic functions is usually the result of such one-pot synthesis. However, excessive proportions of the organosilane adversely affect the structure of the obtained particles, and disordered structures should be expected in many cases when a high degree of organic functionalization is required. Also, organic functions that remain deeply incorporated within the very silica backbone are useless with respect to further derivatization/activation. Moreover, hydrolysis rate of the chosen organosilane can be significantly different form that of the alkyl orthosilicate: therefore, preparations showing heterogeneous distribution of the organic functions could arise.
(iii) Use of the so-called *silsesquioxanes* (general empirical formula R_2_Si_2_O_3_), oligomers derived from hydrolysis—under proper experimental conditions—of organosilanes with general formula X_3_SiR, where X is an easily hydrolysable function such as Cl– or RO– [145]. Bridged organosilanes produce particular silsesquioxanes that could be incorporated within the particle structure by means of a sol-gel method, and later subjected to ammonolysis (with gaseous ammonia) at high temperatures to break one head of the Si–C bonds bridges while inserting –NH_2_ groups on to the organic moieties [146]. The method is promising but requires specialty instrumentation for high-temperature ammonolysis; certain bridged disilanes caused the collapse of the mesoporous structures when subjected to ammonolysis. On the whole, the use of silsesquioxanes (that could also be obtained as polymers of undefined degree of polymerization) is not always well distinguishable from co-condensation.

**Scheme 1:**
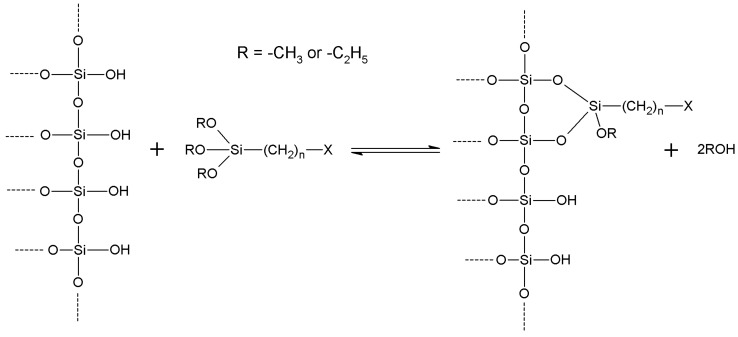
Trialkoxyorganosilanes perform functionalization of silanols on the surface of inorganic supports.

The chemistry of the grafting reaction (“silanization”) has been deeply studied and elucidated mainly in the case of silica-based supports [7,35,147,148,149], but the findings have been extended to other oxides with good results. Silanization (Scheme 1) is usually performed by the means of suitable organosilanes showing the general formula (RO)_3_Si–(CH_2_)_n_–X, where R usually is –CH_3_ or –C_2_H_5_, *n* is 3, and X is a suitable chemical function, useful for subsequent immobilization reactions. However, other organosilanes with different structures are commercially available and could be useful, possibly after further reaction, in immobilization procedures.

On the whole, the silanization reaction consists in a nucleophilic attack of a silanol group on the support to the silicon atom of the organosilane. When a non-silica-based support such as (hydrous) TiO_2_, Al_2_O_3_ or so on is used, a quite similar reaction takes place, and in any case a stable, covalent organosilane coating is formed. A noticeable exception is ZrO_2_ which, under those conditions, is incapable of forming Zr–O–Si bonds resistant against hydrolysis [118].

In the case of silica-based supports, new siloxane bonds, bridging together the matrix and the silane, arise, while methanol or ethanol is given off. The reaction could take place in bulk (without any added solvents, as the common silanes are nonvolatile liquids at room temperature), or in an organic solvent such as toluene or so on [35]. In certain cases (e.g., aminopropyl trialkoxysilanes and analogs), the reaction could also be performed with the organosilane dissolved in water, provided that pH has been lowered by adding a suitable acid to prevent silica support dissolving. Moderate heating (also under reflux) is useful as methanol or ethanol readily volatilize between 60 and 100 °C thus driving the reaction towards completion. In principle, all three alkoxy substituents on the silicon atom of the silane are eliminated upon reaction with the silanol groups on the support surface. Various studies have investigated the intimate mechanism of the silanization reaction, and a widely accepted conclusion is that some alkoxy substituents can survive, owing perhaps to sterical reasons [147,148,150]. However, enzyme covalent immobilization on to silanized inorganic supports usually implies an aqueous environment for preparation and operation of the immobilized catalyst, so the surviving alkoxide functions unavoidably hydrolyze leading to additional silanols, which can in turn condense with each other. The same is true when silanization of an inorganic support is carried with an aqueous solution of the chosen organosilane. In such cases, hydrolysis of the trialkoxysilane takes place, and silanization is achieved upon condensation reactions (leading to siloxane bridges) involving silanol groups (or more generally –OH groups, in the case of non-silica-based materials) of the support and those arising from the above mentioned hydrolysis [151]. It is worth noting that hydrolysis of trialkoxyorganosilanes takes place, to a certain extent, at the expenses of adsorbed water on to support surfaces, also when pure organosilane or its organic solution are used, unless the adsorbed water is not previously eliminated.

The original structure of the plain support is only marginally affected by the grafting, although bulky silanes and/or narrow pores could lead to a substantial obstruction of the mesochannels, when a mesoporous material is used. As grafting takes place exclusively at the surfaces of the support particles, the core structure of the support remains almost unaffected by the modification. Very reactive supports tend to undergo massive grafting, causing an excessive crowding of the functional groups attached to the support. This could cause a substantial wasting of the grafted functional groups, therefore adversely affecting further activation reactions and, on the whole, the yields of immobilization and the catalytic performances of the immobilized enzymes. Moreover, unreacted functions could confer to the support undesired features such as ion exchange properties, when definitely acidic or basic functions are present in the original silane. This is true for example for the most popular agents, namely aminopropyl silanes. As a general rule, “organic” silanization achieves higher silane loadings in comparison to “aqueous” silanization. However, a fraction of bound silane arising from “organic” silanization is lost when the derivatized supports are exposed to water [35].

### 4.2. Grafting the Chosen Functional Group

**Figure 3:**
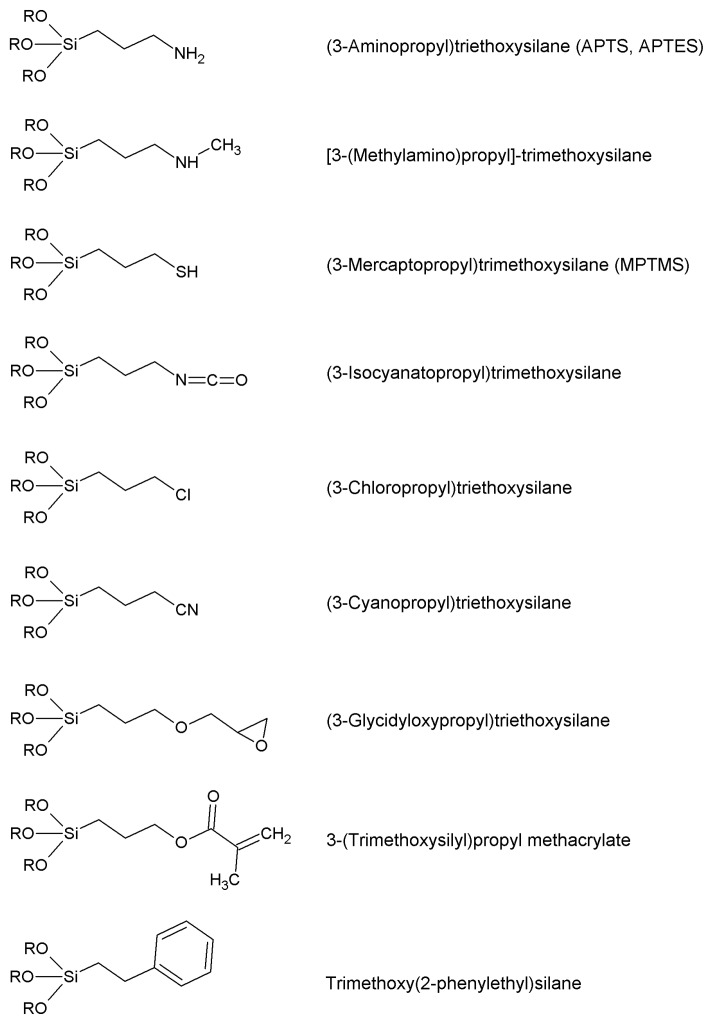
The most widespread organosilanes for the functionalization of inorganic supports during protein immobilization.

The most studied and applied silanes are by far 3-aminopropyltrialkoxysilanes (both the methyl and ethyl alkoxides are widely available at reasonable prices and behave similarly in the silanization reactions). The wide popularity of the aminopropyl function mainly resides in the inexpensiveness and availability of the silanizing agents and in the versatile reactivity of primary amino group in the following activation procedures (§ 5). Aminopropyl trialkoxysilanes (and some commercially available analogues, see Figure 3), besides, are water-miscible and make the functionalized supports hydrophilic, which is often a quite desirable feature for a matrix intended for subsequent enzyme immobilization.

**Scheme 2:**
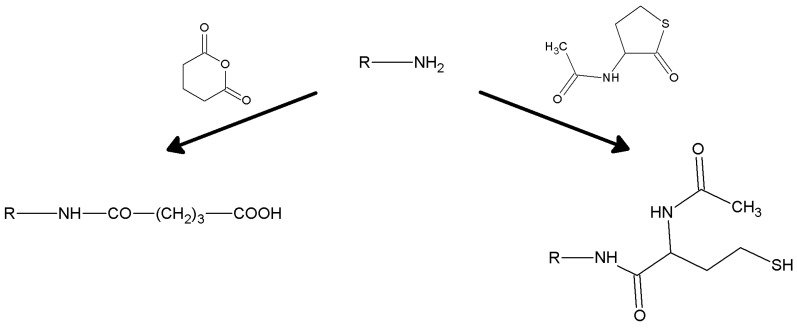
Alkylamine supports can be easily derivatized to carboxyl (using glutaric anhydride, left) or thiol (using *N*-acetyl-DL-homocysteine thiolactone, right) function.

Carboxyl-silanes are not easily synthesized, being not commercialy available at reasonable prices. So, when carboxy functions are required, treatment of an aminopropyl support with glutaric anhydride affords the desired function (Scheme 2) [152]. The reaction typically goes in aqueous suspension of the support, at a nearly neutral pH. One gram of the aminated supports suspended in a fluid slurry in 50 mM sodium or potassium phosphate buffer, pH 6, is treated with 0.3 g of glutaric anhydride in small portions. The pH is kept above 4 by 1 M NaOH; the reaction is complete (as checked with a ninhydrin test [153]) within one hour under gentle stirring. The slurry is then washed carefully to remove the byproduct glutaric acid. Succinic anhydride [78] could also be used, but is less practical as it is only sparingly soluble in water. Glutarylation has the advantage of introducing a hydrophilic bridge between the carrier and the carboxyl function. The carboxyl function could also be inserted on to a carrier by hydrolyzing the nitrile group, previously inserted by means of 3-cyanopropyltrialkoxysilane. Hydrolysis of the nitrile group should be carried out with aqueous H_2_SO_4_, otherwise the use of aqueous NaOH would lead to support dissolving, in the case of a silica-based carrier. The main drawback of this procedure is the shortness of the inserted tether.

Other available and widely used silanes must be cited: among these, glycidyloxypropyl- trialkoxysilanes are peculiar in that they could be the starting points for further chemical transformations, or also can act as activating agents, being capable of directly reacting with enzymes (§ 5.10). The epoxide ring is very reactive; so the silane and the corresponding silanized supports should be treated with the due care to avoid unwanted ring cleavage. Reaction of the functionalized supports with suitable diamines leads—through epoxide ring cleavage—to the insertion of an amino tether, which could be in turn activated for enzyme immobilization. The epoxy function could also be hydrolyzed by aqueous KOH, and therefore a new –CHOH–CH_2_OH moiety is introduced on the support. This can be activated in many ways (§ 5) somewhat mimicking organic polyhydric supports.

Mercaptopropyl-trialkoxysilanes are stenchy liquids, but are useful for direct grafting of the mercaptopropyl moiety onto inorganic supports; however, the resulting materials are sharply hydrophobic, and this feature, together with the limitation of the very short tether, is a serious drawback in the field of enzyme immobilization. The introduction of the thiol function onto a given inorganic support could be however accomplished with a post-grafting modification of an aminopropyl support. The reactive compound *N*-acetylhomocysteine thiolactone (which is an ideal reagent for thiolation of primary amines [154]) under mild alkaline conditions in aqueous environment is capable of inserting a long tether, ending just with the –SH group, while the hydrophilic character of the support is fully preserved (Figure 3).

The commercially available 3-isocyanatopropyl-trialkoxysilanes allow inserting in a given inorganic carrier, the isocyanate function which is reactive towards primary amines under mild conditions. Therefore such silanes could be regarded as activating agents (direct reaction of the isocyanate function with ε-amino groups of lysine residues, *vide infr*a § 5.10). However, the isocyanate-coated carriers are more useful as versatile starting points to obtain a variety of different functionalized carriers, possibly ready for activation [155,156,157,158]. Phenyltrialkoxysilanes as silanizing agents (such as the readily available trimethoxy(2-phenylethyl)silane) lead to a phenyl-coated, hydrophobic supports, that could be nitrated, reduced to the corresponding aromatic amines, and finally diazotized, according to a general procedure of derivatization/activation which starts from a nitro aromatic tether and ends with a reactive diazonium salt (*vide infra* § 5.9). It should be noted that these functionalization procedures also find applications in non-covalent immobilizations, being able, for instance, to modulate the adsorptive properties of silicas [3,30,159].

### 4.3. Catechols as Derivatizing Agents

Titania has the almost unique feature of forming stable complexes with simple catechol (1,2-dihydroxybenzene, *o*-C_6_H_4_(OH)_2_) and with some catechol derivatives, such as DOPA (dihydroxyphenylalanine). This peculiar behavior of titania opens the way to its derivatization, for in principle any catechol derivative, unless excessively crowded with bulky substituents in the *ortho*-position relative to the two phenolic hydroxyls could be used to anchor the functions of choice to titania [160]. Although restricted to titania, the method is promising as functionalization of the support takes place under very mild conditions. Also the inverse approach (covalent attachment of a proper catechol derivative to the enzyme, and subsequent mixing with titania) has been successfully assessed [161] (*vide infra* § 5.9).

### 4.4. The Phosphate/Phosphonate Route

Phosphonic acid (HP(=O)(OH)_2_, previously known as phosphorous acid) is the virtual parent compound of the organic phosphonic acids, where the hydridic hydrogen atom directly bound to the phosphorus is substituted with organic moieties. Phosphonic acids share with orthophosphoric acid and phosphate mono- and di-esters [162] a remarkable affinity towards certain insoluble oxides [163] such as alumina [162,164] and zirconia [165]. In general, *O*-aminoethylphosphate and aminoethanephosphonic acid are ideal agents to introduce primary amine functions on the indicated supports, under very mild conditions (simple soaking of the oxides with dilute aqueous solutions of the reagents). The interaction, which has presumably a partial covalent character, is strong enough to allow obtaining stable immobilized enzymes; however, it can be reverted by treatment with phosphate buffers that form stronger interactions with the supports.

### 4.5. Gold Activation

Gold represents a borderline case when treating covalent functionalization of inorganic supports. Although it is the noble metal by antonomasia, gold shows a noticeable affinity for low-oxidation-state sulfur compounds such as mercaptans (thiols). This feature can be exploited by taking advantage from the facile reaction between gold (nano)particles or films and judiciously chosen mercaptans, bearing an additional function (such as –NH_2_, –COOH, alcoholic –OH, and so on) that could be further modified or activated [134,135]. The (at least) partial covalent character of the gold-thiol interaction is however labile enough to require a very careful manipulation of the obtained preparation to avoid substantial enzyme tether leakage.

## 5. Support Activation and Enzyme Immobilization Techniques

Functionalized supports are usually not capable of reacting directly with proteins. Only epoxy- or aldehyde-functionalized supports for instance can directly couple with enzymes. In all the other cases, the support needs to be activated with specific reagents that ensure reactivity towards protein functional groups. In general, activation of a support implies the insertion of electrophilic functions directly on the support surface. Alternatively, the support is functionalized at first, and in turn the newly introduced functions are chemically modified to change them into the desired electrophilic moieties. In fact, the functions commonly found on the protein surface are nucleophilic, or can be made nucleophilic by a judicious choice of the pH during the coupling reaction. Generally speaking, pH values between 6 and 9 are the most suitable for fast immobilization with high yields, also in terms of enzyme activity retention. Therefore, phosphate-based buffers are the most popular, although more recently some “biological” buffers have been proposed. “Tris” buffers (based on tris(hydroxymethyl)aminomethane) are usually not suitable as their primary amino group can successfully compete with the amino groups of the protein, because of both their higher concentration and lower pK_a_.

Although in principle the higher the pH, the faster and high-yielded the immobilization reaction, one should keep into due account that the hydroxide ions are also nucleophiles; so excessively high pH values on the whole tend to waste a noticeable fraction of the electrophilic moieties on to the support rather than favoring the immobilization. Therefore, the chosen pH should represent the best compromise between the need of preserving the electrophilic power of the support and the required good nucleophilic character of the target functions on the protein. When working on comparatively unreactive electrophilic carriers, also dilute NaHCO_3_ solutions (pH = 8.3) could be the solvent of choice to obtain excellent results. On the contrary, very reactive carriers behave at their best performance at pH ≈ 6, where a little but sufficient concentration of un-protonated lysine residues is still present, but in certain cases even lower pH are suitable.

The most targeted protein function is definitely –NH_2_, both *N*-terminal α-amino group and lysine ε-amino group. Several reasons explain that: (*i*) it is widespread in almost all proteins; (*ii*) reactivity is optimal; (*iii*) lysine has usually a minor mechanistic relevance (so it is not fundamental for retention of catalytic activity). However, in some cases protonated –NH_3_^+^ can be involved in ionic interactions crucial for the stability of proteins: in these cases, their engagement in linkages with the support could instead lead to denaturation; (*iv*) it is usually present at protein surface [166], being therefore easily accessible during coupling reactions [167].

Lysine ε-amino groups are, however, quite basic, allowing their presence in non-dissociated form only at alkaline pH values (>9–10), not always compatible with protein stability. Otherwise, the reactivity of protonated –NH_3_^+^ is negligible. A possible solution could be chemical amination of proteins, inserting more reactive amino functions [168]. An α-amino group, on the contrary, has a lower pK_a_ (≈7–8), extending its effectiveness in nucleophilic attack almost to neutrality [33], but is not always exposed at the protein surface. Fortunately, as noted above, also at pH ≈ 6 the very low percentage of un-protonated lysine residues can be sufficient to couple with very electrophilic carriers.

Carboxylic groups are also quite abundant [166,168], but they are usually not reactive in the typical immobilization protocols, needing further activation [169]. Other potentially useful groups are the nucleophilic alcoholic (serine, while threonine is usually unreactive [166]), phenolic (tyrosine), imidazole (histidine), and thiol (cysteine) groups, but their lower frequency prevents widespread use. In particular, it is common knowledge that histidine is a relatively rare aminoacid in proteins, and moreover when present is usually engaged in more or less specific linkages with other aminoacids residues: therefore, its (low) nucleophilic power is, as a rule, not enough to make the aminoacid a candidate for binding the enzyme to a support.

In this section, we review the most common approaches towards activation of supports and subsequent protein coupling (summarized in Table 2). Particular focus has been put on molecular mechanisms and operational conditions of the reactions. Operators should take particular care of the molecular spacer inserted by activation between protein and carrier, since it can specifically tune catalytic features of immobilized protein. It has been observed that short spacers lead to rigidification of protein structure, and therefore to its stabilization [8]. On the other hand, when long molecular spacers are used, the native protein structure is less affected, thence preserving the protein native structure. Moderate stiffening of tertiary (and possibly quaternary) protein structures very often leads to substantial stabilization, which is therefore observed in most immobilized enzyme preparations. However, multipoint enzyme coupling (*i.e.*, the same enzyme molecule is engaged in several covalent linkages to the support) stabilizes the protein structure to the point that the reversible conformational changes taking place along every catalytic cycle become difficult or also impossible, therefore leading to almost inactive enzyme preparations, so an excessive crowding of electrophilic functions onto a carrier surface usually leads to poorly active preparations, owing to excessive crowding of the immobilized molecules and subsequent reciprocal interference and hindering, and also to the above-mentioned multipoint attachment.

### 5.1. Cyanogen and Cyanuric Halides

Cyanogen and cyanuric halides can be used to activate both plain silicas (using silanol functions) and –NH_2_ functionalized supports. Cyanogen bromide (BrCN) is the most widespread halide for this application, despite its volatile, lachrymatory, toxic and explosive nature. Cyanogen chloride ClCN is even more toxic and dangerous; cyanogen iodide ICN is costlier, and none of these presents any advantage compared to BrCN. This mode of immobilization *per se* forms a very short molecular spacer between the support and immobilized protein, unless an additional spacer has previously added to the carrier.

**Table 2:** Summary of the most common methods of activation for inorganic supports.

Activation Method	Support Reactive Group	Protein Reactive Group	Type of Bond	Bond Stability	Cost of the Reagents	Molecular Spacer
**Cyanogen bromide**	-OH -NH_2_	-NH_2_	Isourea or imido-carbonate	Low	Moderate	Very short
**Cyanuric chloride**	-OH -NH_2_	-NH_2_	Secondary amine	High	Low	Medium length
**Sulfonyl halides**	-OH	-NH_2_ -SH	Secondary amine or thioether	High	Moderate/high	None
**Acyl halides**	-OH	-NH_2_	Carbamate	High	Moderate/high	Very short
**Thionyl chloride**	-COOH	-NH_2_ -SH	Amide/thioester	High	Low	None
**Metal halides**	-OH	-SH	Metal bridge	Moderate	Moderate	Very short
**Glutaraldehyde**	-NH_2_	-NH_2_	Secondary amine	High	Low	Long
**Carbodiimides**	-COOH/ -NH_2_	-NH_2_/ -COOH	Amide	High	High	None
**Divinylsulfone**	-OH -NH-	-SH -NH_2_	Ether/Secondary amine/thioether	Good (at neutral pH)	Moderate	Medium length
**Benzoquinone**	-OH -NH_2_	-NH_2_ -SH	Anilinyl	High	Low	Medium length
**Disuccinimidyl suberate**	-NH_2_	-NH_2_	Amide	High	High	Long
**Succinimidyl-4-(*N*-maleimidomethyl)cyclohexane-1-carboxylate**	-NH_2_	-SH	Amide Thioether	High	High	Long
**2-2'- and 4,4'-Dipyridyldisulfide**	-SH	-SH	Disulfide	Moderate	High	Very short
**1,6-Bismaleimidohexane**	-SH	-SH	Thioether	High	High	Long
**Carbonyl diimidazole**	-OH	-NH_2_	Carbamate	Moderate	Low	Very short
**Diazotization**	Aromatic-NH_2_	Aromatic -OH	Azo bond	High	Moderate	Medium
**Epichlorohyridin**	-OH -NH_2_	-NH_2_	Secondary amine	High	Low	Short

In alkaline environment (typically NaOH, but a moderate basicity—pH 10/11—by tertiary amines results in even higher yields [170]), the support and BrCN are slowly suspended in water with a 4:1 *w/w* ratio [35]. Temperature is kept at 4 °C for 30’. BrCN can be previously dissolved in a water-miscible solvent (not reactive with BrCN) such as cold acetone.

**Scheme 3:**
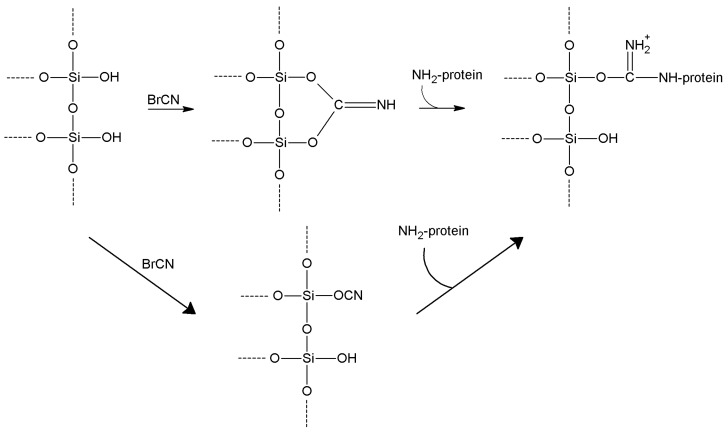
Possible mechanism for cyanogen bromide activation of silanol functions [35,173,174].

After exhaustive washes with chilled water, the support is ready for coupling with protein amino-groups. In fact, a cyanic acid ester, very reactive and unstable, has been formed, presenting a highly electrophilic carbon atom, and spontaneously changing into a imidocarbonate [171], which is still reactive towards lysine residues. Thus, lysine ε-NH_2_ is very prone to nucleophilic attack, forming an isourea (or imido-carbonate) bond (Scheme 3). Unfortunately, this mode of immobilization is often not very stable towards hydrolysis [172,173,174], as it contains the rather labile Si–O–C bridge; reasonably stable immobilized preparations are however obtained in the case of multipoint enzyme attachment.

**Scheme 4:**
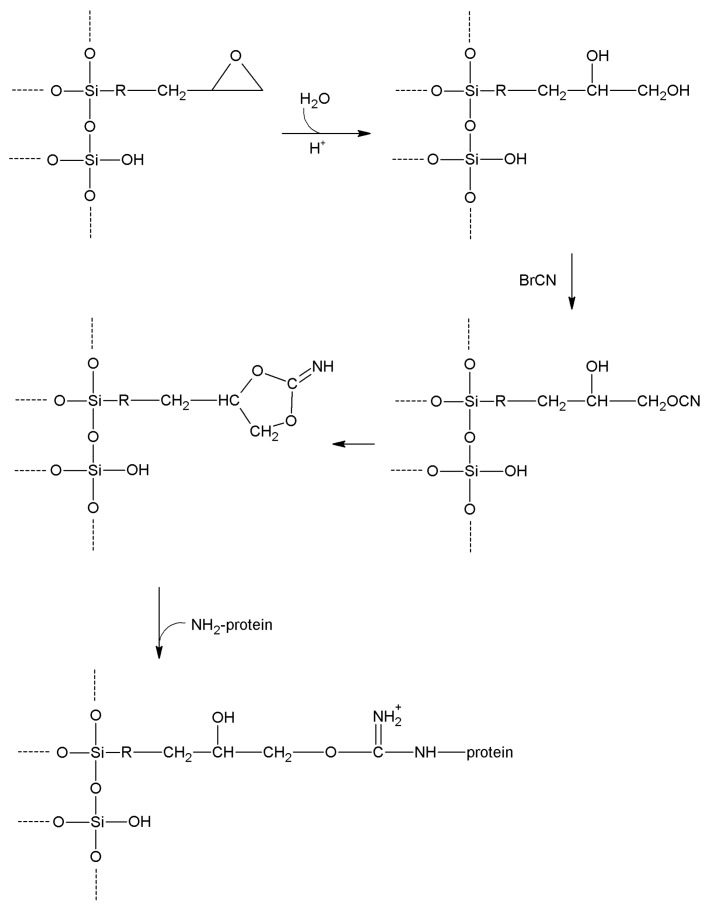
Reaction pathway for cyanogen bromide activation of epoxy-functionalized supports.

More stable immobilization could be achieved when alcoholic functions have been introduced into an inorganic carrier; this can be accomplished by grafting 3-glycidyloxypropyl trialkoxysilane on to the support, and then hydrolyzing the epoxy function by hot dilute HCl [175]. A 3-glyceryloxypropyl function arises, ready to react with BrCN (Scheme 4).

**Scheme 5:**
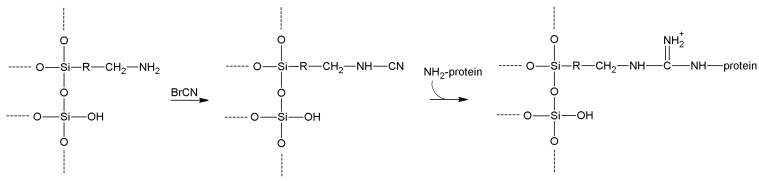
Reaction pathway for cyanogen bromide activation of amino-functionalized supports.

More stable interactions occur when—as originally proposed [176]—BrCN is used to activate aminated supports such as aminopropylsilica carriers (Scheme 5). In such cases, relatively stable cyanamide functions are inserted on the support, that are in any case reactive enough to bind lysine side chains, leading to stable immobilization through hydrolysis-resistant and charge-retaining substituted guanidines [125].

**Figure 4:**
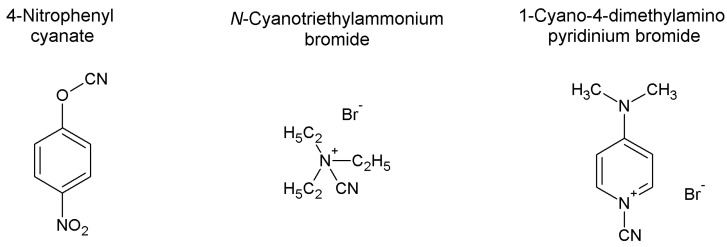
4-Nitrophenyl cyanate, *N*-cyanotriethylammonium bromide, and 1-cyano-4-dimethylaminopyridinium bromide have been described as effective cyanylating agents in alternative to BrCN [177].

Other cyanylating agents have been proposed to overcome the typical drawbacks of BrCN approach (toxicity and immobilized enzyme leaching, particularly). Namely, 4-nitrophenyl cyanate, *N*-cyanotriethylammonium bromide, and 1-cyano-4-dimethylaminopyridinium bromide have been described (Figure 4) [177]. Using a quite similar protocol, support and cyanylating agent are suspended in water or in 0.2 M aqueous triethylamine (only for 4-nitrophenyl cyanate, also requiring the presence of acetone as the reagent is water-insoluble) at 4 °C for a few minutes. After exhaustive washings with ice-cold water, the activated support is ready for coupling. 

**Scheme 6:**
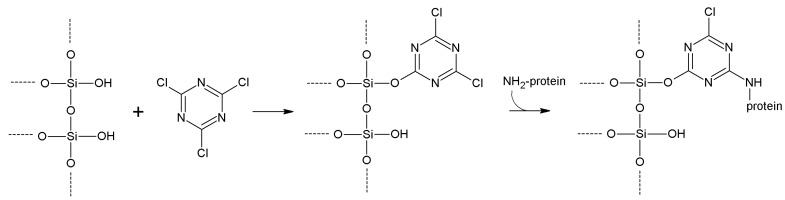
Activation of silanol functions with TCT.

Cyanuric chloride (2,4,6-trichloro-1,3,5-triazine, TCT), which however is not a true cyanylating agent, is often treated together, as it can activate both –OH and –NH_2_-containing carriers. It is the cyclic trimer of cyanogen chloride, being quite less toxic and less expensive than BrCN. Electron-poor carbon atoms of TCT can undergo nucleophilic attack from both silanol –OH, and –NH_2_ of amino-functionalized silicas or other supports (Scheme 6). Lysine ε-NH_2_ then performs the same substitution reaction towards another carbon atom of TCT. More stable secondary amine bond is formed, and a slightly longer molecular spacer is inserted between carrier and protein.

At 4 °C, 1 g of support is suspended in aqueous buffer or in organic solvent (e.g., acetone or dioxane) mixture with 0.15–1.5 g cyanuric chloride [178,179]. TCT is almost insoluble in water but soluble in a wide range of organic solvents; Moreno and coworkers found 0.15 g of cyanuric chloride dissolved in toluene per gram of support as the best conditions for subsequent lipase immobilization [180], leading to a 37 times more stable enzyme with 80% residual activity after 336 h of operating time. After at least 3–4 h and exhaustive washes at first with acetone then with buffer, the activated support is ready for protein coupling (3 h, 4 °C [180]).

### 5.2. Sulfonyl Halides

**Figure 5:**
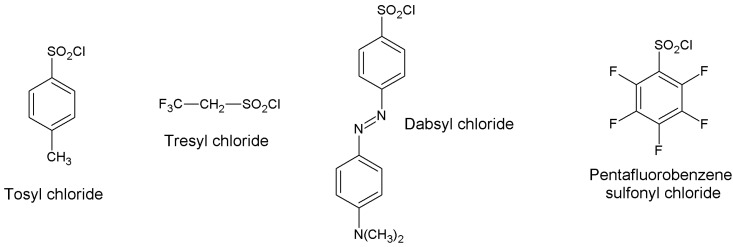
The most common sulfonyl halides used in protein immobilization.

Sulfonyl halides (in practice, only the chlorides R-SO_2_Cl are commonly used) are able to convert alcohols into the corresponding sulfonyl esters, which in turn act as alkylating agents towards several nucleophiles [181]. So, they are convenient reagents also in the activation of inorganic supports for protein immobilization. Several sulfonyl chlorides have been proposed in this context. Among these, the highly toxic, reactive, moisture-sensitive and expensive triflyl chloride CF_3_SO_2_Cl give rise to triflate esters so reactive that they largely hydrolyze along the immobilization procedure. Tosyl chloride *p*-CH_3_C_6_H_4_SO_2_Cl is much more handy and quite inexpensive, but its low reactivity is a noticeable flaw during the immobilization procedure. The most used is probably tresyl chloride [182] CF_3_CH_2_SO_2_Cl which, although costly, has the ideal reactivity for activation procedures. Nevertheless the colored dabsyl and the reactive pentafluorobenzene sulfonyl chloride have been reported (Figure 5) [181,183].

**Scheme 7:**
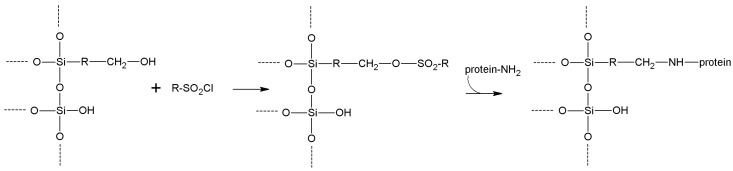
Activation of primary alcoholic functions by sulfonyl halides.

Among nucleophiles, primary alcohols are the functions of choice in this perspective, whereas silanols poorly react [136,181]. Secondary alcohols are also sulfonylated, but the corresponding esters are unreactive towards nucleophiles, owing to steric hindrance. Primary amines could also be sulfonylated by sulfonyl chlorides, but the resulting sulfonamides are quite inert and therefore useless. As depicted in Scheme 7, in an anhydrous and alkaline environment, primary alcohols perform a nucleophilic attack towards electron-poor sulfur atom. An enough reactive sulfonic ester is formed, while HCl is released (accounting for the need of a proper base—*i.e.*, pyridine, triethylamine, *N*-methylmorpholine or so on—to neutralize it). In a second step, –NH_2_ functions from proteins can in turn bring nucleophilic attack to activated support, yielding stable secondary amine linkage (or thioether, in case of cysteine performing final nucleophilic attack) [184]. The sulfonyl group is released in solution as the corresponding sulfonate anion, which allows quantifying the immobilization yield when a colored activating compound such as dabsyl chloride has been used.

Whereas tresyl chloride affords almost quantitative yields of immobilization at neutral pH and 4 °C, tosyl chloride requires alkaline pH (>9) [181]. Such a behavior can be explained taking into due account the higher electron-withdrawing power of the CF_3_CH_2_– moiety in comparison with *p*-CH_3_C_6_H_4_–. In anhydrous organic solvent (*i.e.*, acetone or dioxane) the alcoholic –OH functionalized support is suspended in the presence of equimolar sulfonyl chloride/organic base (such as pyridine) mixture at 4 °C for 15–30’. 50–150 µmol sulfonyl groups per gram of support can be inserted with nearly theoretical yields [181,185]. Direct activation of titania –OH has been reported to require longer reaction times (about 48 h, 37 °C, using tresyl chloride [136]). After exhaustive washings with organic solvent and water, the activated support can be immediately used for coupling, or dried and stored in desiccator. Protein coupling then occurs at 4 °C and pH almost neutral for several hours [181].

### 5.3. Other Acyl Halides and Analogues

**Figure 6:**
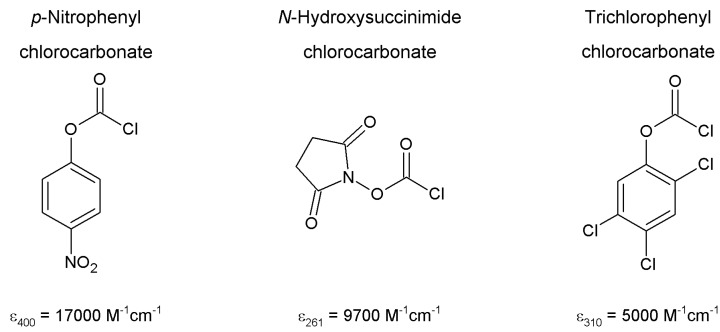
The most used chlorocarbonates for the activation of hydroxyl-bearing supports. The molar extinction factor of the leaving group is reported.

Some acyl halides have also been described as effective activating agents for –OH functionalized supports. The most useful identified have been two chlorocarbonates: 4-nitrophenyl chlorocarbonate and *N*-hydroxysuccinimide chlorocarbonate (Figure 6), while some trichlorophenyl chlorocarbonates have been also used with lower efficiency [186,187].

**Scheme 8:**
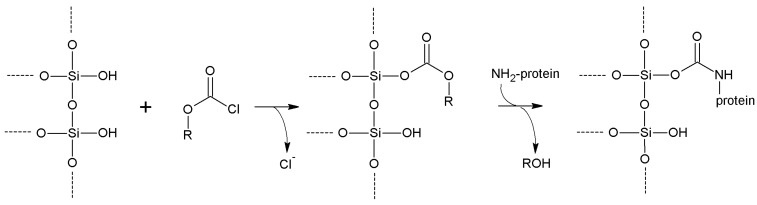
Mechanism of activation using chlorocarbonates.

When reacting in organic and alkaline suspension with –OH (both silanols and alcoholic), such reagents give activated carbonates in sufficient yields (15%–25% [187]), with chloride ion as the leaving group. Subsequently, the activated support can react with protein –NH_2_ groups (Scheme 8). The coupling reaction gives a relatively stable carbamate linkage, with almost complete functionalization of all the accessible –OH groups [187]. Carbonic acid mixed esters arise, whose leaving groups upon reaction with the enzyme usually have absorption maxima in UV or visible range, enabling spectrophotometric check of the activation.

During activation, an organic base (such as triethylamine, pyridine or so on [186]) is required to neutralized the released HCl. In the typical protocol, the support is suspended in anhydrous acetone with a proper amount of the chosen chlorocarbonate (highest yields were observed with 100 µmol chlorocarbonate per gram of support [187]). The organic base is then added with a molar ratio of 1.5–2. After 1 h at 4 °C, the activated support is exhaustively washed with acetone, and can also be stored for several months at 4 °C in 2-propanol. Activation can be quantified by alkaline hydrolysis, and spectrophotometric determination of colored leaving groups (molar extinction factors are reported in Figure 6).

Protein coupling occurs at slightly basic pH (≈7.5–8.5). In ice-cold buffer, several hours of reaction are required (up to 16 for *N*-hydroxysuccinimide chlorocarbonate, 48 h for 4-nitrophenyl chlorocarbonate). Unreacted active carbonate groups can be eventually removed by alkaline hydrolysis (aqueous ammonia or dilute NaOH can be used, taking into the due account their compatibility with protein stability), if their presence possibly interferes with the application of the immobilized enzyme [187].

Minor leaching issues are associated with these chlorocarbonates (if compared, for instance, with BrCN), while reagents with lower toxicity are employed. No additional charges are inserted by carbamate bond, and the possibility of remove the residual reactive groups from support stands as an additional advantage of this technique.

**Scheme 9:**
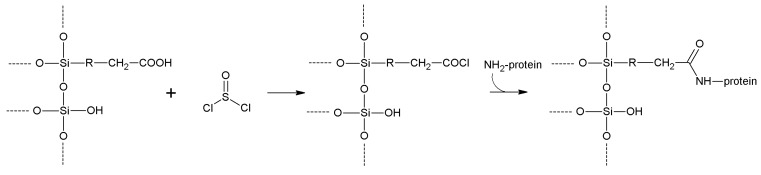
Mechanism of activation using thionyl chloride.

Another possible approach involving acyl halides functions envisages the use of thionyl chloride (SOCl_2_) [29]. This reactant in anhydrous environment activates –COOH functions to acyl chlorides –COCl (4–6 h, in chloroform for instance [29]), which in turn couple with protein –NH_2_ and –SH (Scheme 9). Activation requires a slight alkaline pH (about 8.5) and about 2 h [188]. Unfortunately, the concomitant fast hydrolysis in water of the acyl chloride intermediate prevents high yields with this technique. For activation of poorly reactive carboxy carriers, such as aromatic acid functions, PCl_5_ could replace thionyl chloride.

### 5.4. Metal Halides

**Scheme 10:**
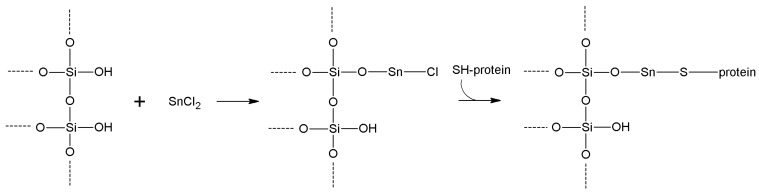
The proposed mechanism for metal bridge activation of silanols [60].

Metal halides have also been described to directly activate –OH functions [60]. TiCl_4_, TiCl_3_, SnCl_4_, SnCl_2_, ZnCl_4_, VCl_3_, FeCl_2_, and FeCl_3_ have been the most successful, but the best results among them have been observed using SnCl_2_ [35]. The exact mechanism of this reaction is still unknown, but some evidence suggests a likely partial covalent nature of the interaction, and not a simple adsorption, as reported in Scheme 10. For certain, the strength of the resulting protein/carrier interaction is higher than simple adsorption. Besides, increased stability of immobilized protein has been described [60].

Each gram of support is suspended in 40 mL of 1% SnCl_2_. After 45’ at 37 °C and exhaustive washings with water or buffer, the activated support is ready for protein coupling at pH close to neutrality [35]. Alternatively, the activated support can be also dried and stored for later coupling [35]. Cysteine –SH functions seem to be involved in the formation of metal bridge.

### 5.5. Glutaraldehyde

Glutaraldehyde OHC-(CH_2_)_3_-CHO is a very popular bifunctional agent in protein cross-linking and covalent immobilization, needing –NH_2_ functions both in carrier and in protein. Concentrated glutaraldehyde aqueous solutions (25% or 50%) are readily available and inexpensive, and the procedure is quite simple, accounting for the wide success of this method [189].

Since the reaction involves a bifunctional aldehyde and aminated molecules, the formation of Schiff base between glutaraldehyde and support (firstly), and glutaraldehyde and protein (later) is often mistakenly described. However, much evidence suggests otherwise. (*i*) The formed bond is typically irreversible; (*ii*) Only glutaraldehyde gives such reaction, whereas similar bifuctional aldehydes (such as malonic or succinic) poorly react; (*iii*) Along the reaction, the support becomes intensely colored; (*iv*) HCl 6 M at 110 °C by 24 h does not hydrolyze bonds between glutaraldehyde and proteins [190]. It has been shown that commercial solutions of glutaraldehyde contain at least 13 different forms (depending on pH, concentration, and temperature), including oligomers that can evolve to α,β-unsaturated aldehydes after dehydration [191].

**Scheme 11:**
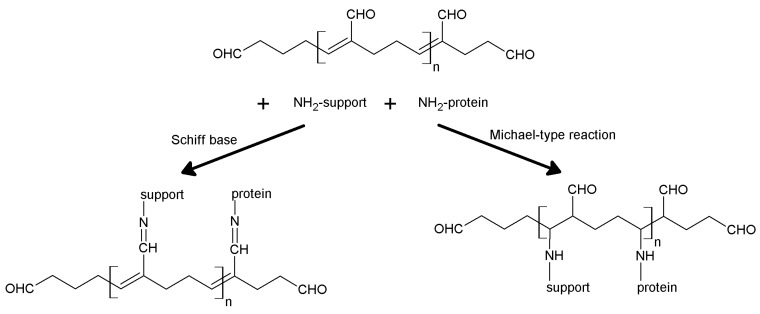
Glutaraldehyde can theoretically react with –NH_2_ groups through two distinct mechanisms: Schiff base and Micheal-type addiction. However, the second is by far the most plausible [190,191].

Accordingly, several mechanisms have been proposed instead of Schiff base formation. The most accepted involves Michael-type addiction of –NH_2_ to α,β-double bonds, yielding a stable secondary amine (Scheme 11). More rare heterocyclic forms of glutaraldehyde could be responsible for the development of color during reaction. However, the exact mechanism still remains unknown [190,191].

In the typical protocol, the reaction occurs in neutral (using phosphate buffer,* i.e.*,) 2.5% glutaraldehyde solution containing 1 g of support per 100 mL of solution. After at least 60’, the same phosphate buffer is used for exhaustive washings. A semi-quantitative evaluation of the activation degree could be estimated by observing the color change of the support to yellow (low activation) to orange (moderate) to reddish brown (strong). Then, the activated support is ready for protein coupling at pH 6–8 [35]. At these pH values, the most reactive function is protein terminal –NH_2_ (pK_a_ ≈ 7–8), rather than lysine ε-NH_2_ (pK_a_ ≈ 10) [33]. As a matter a fact, this is one of the few activation procedures occurring also at slight acidic pH [29].

An important advantage of this method is the insertion of a long molecular spacer between support and protein, minimizing steric hindrance issues. On the other side, glutaraldehyde-activated supports are versatile multifunctional carriers, being able to give at least two kind of interactions (ionic exchange and hydrophobic adsorption) in addition to covalent linkage [33]. Therefore, when covalent immobilization is required, non-specific weak interactions should be carefully ruled out (*i.e.*, using cationic detergents) [192]. To this purpose, lowly activated supports should be preferred (*i.e.*, using low amounts of glutaraldehyde per support amino-functions) [33]. Unfortunately, this kind of approach is very slow and does not allow multipoint attachment [7]. In some cases, however, the multifunction of highly glutaraldehyde-activated supports can be a positive feature, since the resulting complex mixtures of immobilization mechanisms (covalent, hydrophobic adsorption, ionic exchange for instance) result in strong activation and stabilization of enzymes [33]. Strict control of the desired or undesired mechanisms of immobilization is also possible, modulating for instance ionic strength or presence of detergents to prevent unwanted interactions, and promote the desired ones. Improvement of stability and catalytic features using glutaraldehyde coupling has been described for several enzymes, such as proteases [193], oxidases [194], lipases [195].

### 5.6. Carbodiimides- and Active-Esters-Based Methods

**Scheme 12:**
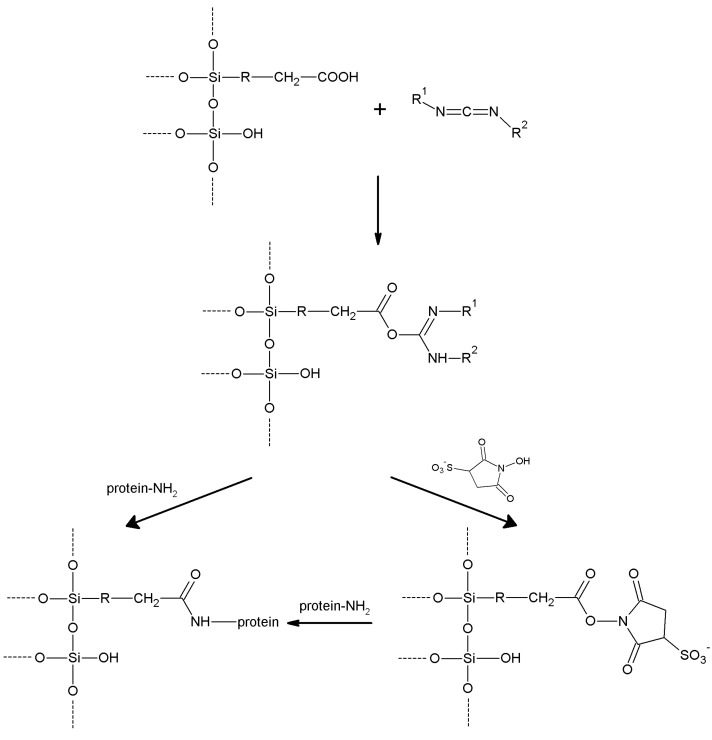
Carbodiimides activate carboxy-functionalized silicas, both in presence or absence of sulfo-*N*-hydroxysuccinimide [196].

Carbodiimides are quite costly reagents able to activate –COOH functions, and make them reactive towards nucleophilic groups (such as lysine ε-NH_2_). Several carbodiimides have been synthetized, but the most popular is 1-ethyl-3-(3-dimethylaminopropyl)carbodiimide (EDC or EDAC) [196]. The central carbon atom of EDC is quite electron-poor, and thus it is quite susceptible to nucleophilic attack by –COOH from support (Scheme 12). An amine-reactive *O*-acylisourea is formed: the still electron-poor EDC carbon atom does enhance the electron deficiency on the carbon atom of the carboxy function. This is therefore able to directly undergo nucleophilic attack by protein amino groups. However, isourea ester intermediates are quite unstable and prone to hydrolysis. The presence of* N*-hydroxysuccinimide (or the more water-soluble analogue sulfo-*N*-hydroxysuccinimide) converts the intermediate to a more stable (but still amine-reactive) *N*-hydroxysuccinimide ester, stabilizing it (in fact, the active succinylated support is commercially available in this form). Coupling efficiency is enhanced by up to 20-fold [197,198]. 1-Hydroxybenzotriazole can be used instead of *N*-hydroxy-succinimide [199].

In a slightly acidic buffer (pH 5/6.5), –COOH functionalized support is suspended with carbodiimide and sulfo-*N*-hydroxysuccinimide and kept under gentle stirring at 4 °C for 30–120’ [200]. Usually a molar ration 1:1:2.5 –COOH functions:carbodiimide:* N*-hydroxysuccinimide is used [201]. Enzyme solution is then directly added to activated support for coupling.

By inverting the order of reactions, carbodiimides can also cross-link –NH_2_ functions from support and –COOH groups from enzyme [2]. However, the presence of Asp and/or Glu residues within the active site of the enzyme could easily lead to irreversible inactivation. No molecular spacer between carrier and protein is inserted by this method, which is however one of the few suitable for proteins unstable at alkaline pH values [29].

### 5.7. Other Bifunctional Agents

In addition to those above described, several other bifunctional agents have been described for protein immobilization. Among them, divinylsulfone has been definitely one of the most employed, mainly because of its stability in water, high yields under mild operational conditions, and stability of the bond between protein and support [167]. Besides, no particular byproducts are generated during activation.

**Scheme 13:**
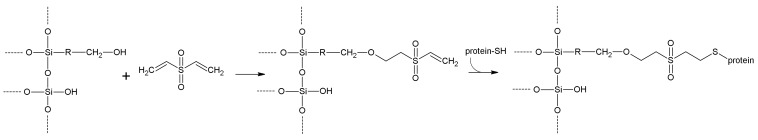
Divinylsulfone is able to activate mainly alcoholic –OH-modified silicas, allowing coupling with cysteine –SH functions [167].

The electron-withdrawing effect of sulfone function activates the vinyl double bond towards Michael-type addition by a wide range of nucleophiles. Primary amines react even too easily, possibly leading to unwanted support reticulation. Accordingly, secondary amines (*i.e.*, from silanization with [3-(methylamine)propyl]-trimethoxysilane) or primary alcohols (*i.e.*, from H_2_SO_4 _treatment of epoxy-functionalized carriers) are preferred (compare § 4.2) [167,202]. Cysteine –SH groups are then the principal residues involved in protein coupling (Scheme 13) [37]. Also lysine ε–NH_2_ has been reported to react with divinylsulfone-activated supports, but the reaction is slow and incomplete, requiring besides alkaline pH values (>9), not always compatible with enzyme biological activity [203]. C–N is however a rather more stable bond than C–S [37].

Both in alkaline aqueous buffer or organic solvent (*i.e.*, tetrahydrofuran and 2-propanol), 5%–10% solution of divinylsulfone are allowed to react with the chosen support for a period of time depending on reactivity of functionalizing functions (2–16 h, room temperature) [139,167,202]. The alkalinity of the buffer should not exceed 9–11 (carbonate is, for instance, a good choice [202]). Over-reaction should be avoided, since unwanted cross-reticulation is possible. After exhaustive washes, protein coupling can be performed in about neutral buffer at 4 °C for several hours, until the desired degree of immobilization has been reached.

The formed bond is quite stable, at least at acid/neutral pH values [37]. A quite long (five atoms) molecular spacer is inserted between protein and carrier, leading to preservation of enzymatic activity (for instance, in the case of invertase a specific activity about 2.5 times larger has been reported [204]).

**Scheme 14:**
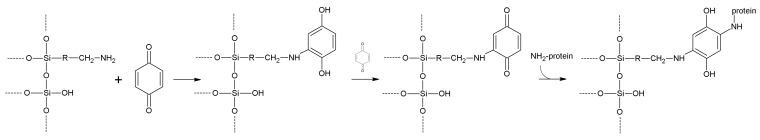
Mechanism of activation using *p*-benzoquinone.

Vinyl functions also undergo Michael-type additions when adjacent to a carbonyl, such as in the case of *p*-benzoquinone [37]. Thus, this reactant can be used in alternative to divinylsulfone, also allowing a long molecular spacer. Both alcoholic –OH and –NH_2_ functionalized support can be used in this technique, yielding an activated support able to couple mainly with –NH_2_ and –SH groups from proteins (Scheme 14). Activation occurs in a wide range of pH values (about 3–10 [37]) with satisfactory yields and in quite short time. In fact, functionalized support requires just 1 h of gentle stirring with 0.01–0.1 M *p*-benzoquinone solution (in ethanol 20% *v/v*) [205,206]. After exhaustive washings with buffer, the activated support is ready for coupling (variable period of incubation are reported, 0.5–24 h [37,205,207]). A quite promising stabilization has been reported using this type of activation [205].

Thiophosgene (CSCl_2_) is another bifunctional agent able to activate aminated-supports with isothiocyanate functions (R-NCS), which in turn can couple with protein nucleophiles [29]. Activation requires 4–12 h in organic solvent (chloroform,), while coupling occurs at slightly alkaline pH (≈8.5). Unfortunately, this reagent is very toxic and the activated support is quite unstable [29].

Isothiocyanate functions can be inserted also with more manageable bifunctional agents [208]. In a recent paper, Aissaoui and coworkers compared for instance several aromatic bifunctional cross-linkers, such as terephtalaldehyde, 1,4-phenylenediisocyanate, 1,4-phenylenediisothiocyanate, [209]. Amino silica was activated in organic solvent (according to solubility of each-crosslinker) for 2 h, then washed and coupled with the protein. 1,4-Phenylenediisocyanate and 1,4-phenylenediisothiocyanate gave the best operational activity and thermal stability during immobilization of glucose-6-phosphate dehydrogenase, showing that the geometry of the cross-linker (*i.e.*, *meta*- or *para*-orientation) changed the residual activity significantly. In fact, *para*-analogues resulted in a decreased interfacial steric hindrance.

**Scheme 15:**
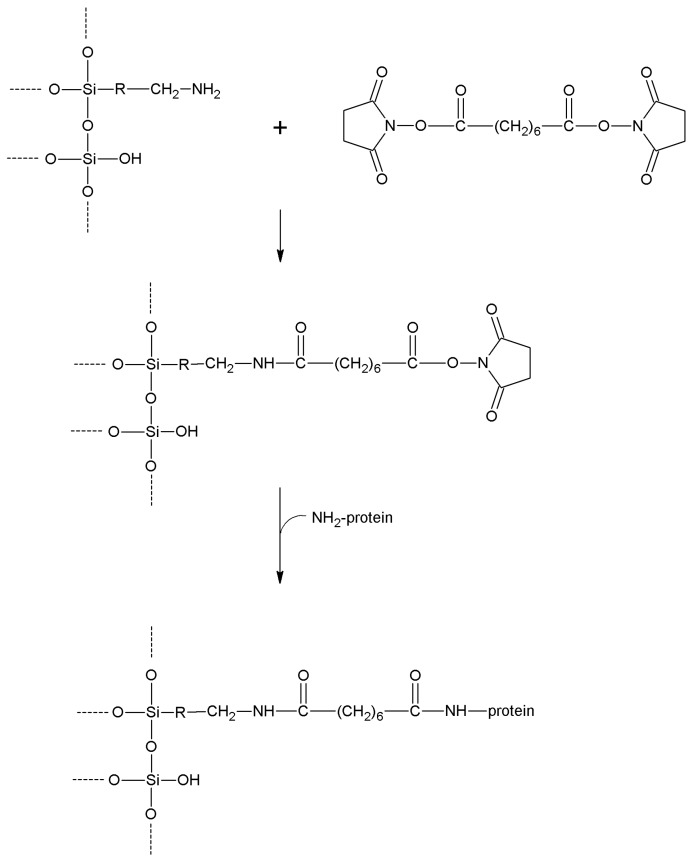
Mechanism of activation using disuccinimidyl suberate.

Amino groups can be also activated using several bifunctional *N*-hydroxysuccimide esters, containing different spacers between the two reactive groups (*i.e.*, suberate and glutarate are commercially available). For instance, *N,N'*-disuccinimidyl suberate can activate alkylamine inorganic supports, introducing a quite long molecular spacer, and then couple with protein –NH_2_ (Scheme 15). The resulting amide bonds result in a quite good stability.

Disuccinimidyl suberate is dissolved in anhydrous organic solvent (*i.e.*, methanol or dimethylformamide) and mixed with a ratio 1:10/1:5 *w/w* with –NH_2_ functionalized support [210]. After 60’ stirring and exhaustive washes with organic solvent, coupling with protein is performed in slightly alkaline buffer [35]. About 1–3 h of reaction are usually necessary to reach satisfactory yields [210]. *N*-succinimide-activated support can be alternatively dried and stored in desiccator at room temperature for several months with negligible loss of active groups [210].

**Figure 7:**
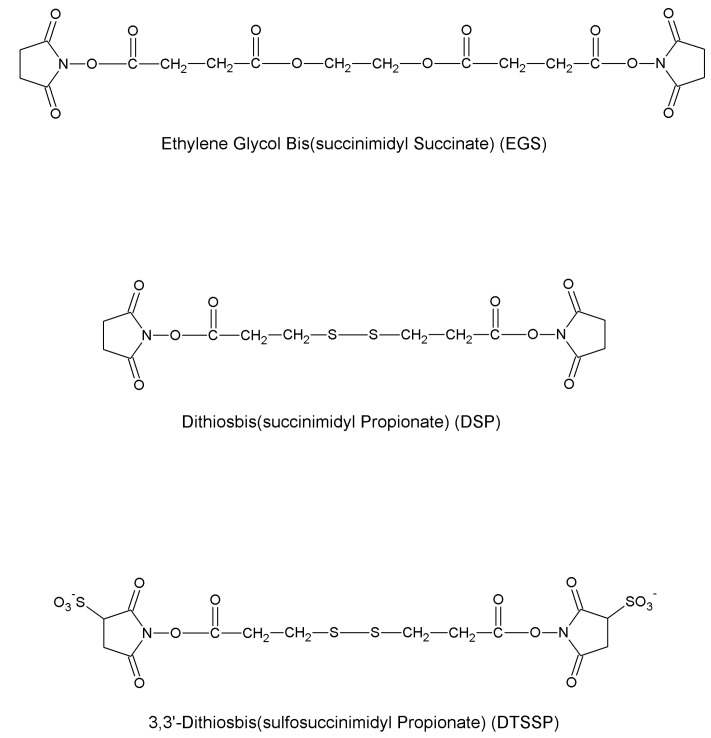
Several bifunctional *N*-hydroxysuccinimides esters containing cleavable cross-linking have been described.

Other bifunctional *N*-hydroxysuccimide derivatives are commercially available with molecular spacers containing cleavable bonds. They are particularly useful for reversible immobilizations enabling the recovery of costly supports after inactivation of the catalyst. The most widespread are reported in Figure 7. 

The procedure of immobilization is the same as above, but for instance ethylene glycol *bis*(succinimidyl succinate) (EGS) immobilized proteins can be released with mild hydroxylaminolysis (but with quite low yields [211]). Significantly better performing releases can be obtained with disulfide-containing crosslinkers (such as dithiobis(succinimidyl propionate) and its hydrosoluble analogue 3,3'-dithiobis(sulfosuccinimidyl propionate), Figure 7), using reducing agents like dithiothreitol [211].

**Scheme 16:**
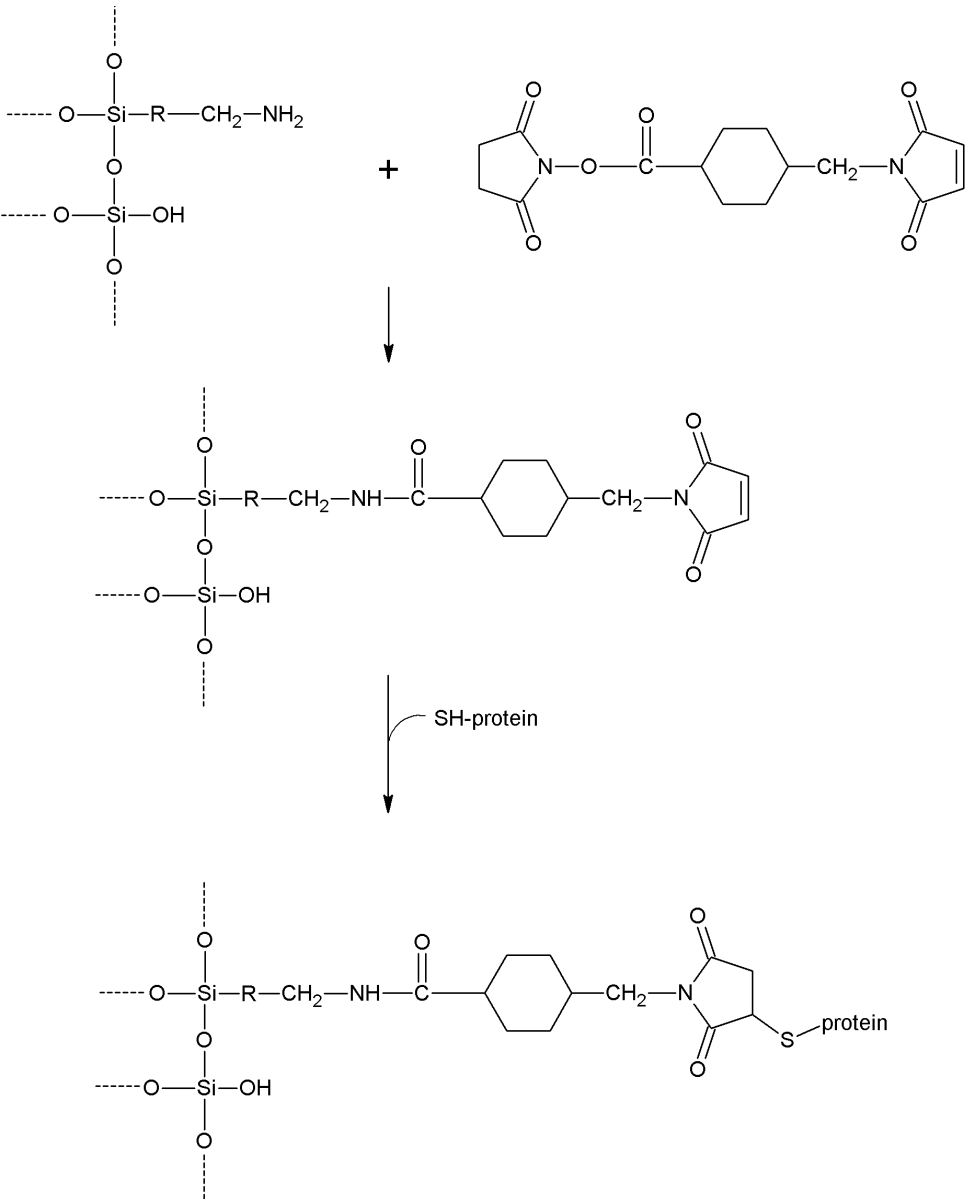
Mechanism of activation using succinimidyl-4-(*N*-maleimidomethyl)cyclohexane-1-carboxylate (SMCC).

When lysine residues are crucial for protein activity or stability, bifunctional crosslinker are commercially available bearing *N*-succinimidyl moiety on one side, and *N*-maleimidyl on the other. For instance *N*-succinimidyl-3-maleimidopropionate (SMP) or succinimidyl-4-(*N*-maleimidomethyl)cyclohexane-1-carboxylate (SMCC) activate alkylamine supports as seen above, but the coupling occurs with protein –SH, as reported in Scheme 16. Protein thiols perform a nucleophilic addiction to α,β-double bond of *N*-maleimidyl moieties, yielding a stable thioether linkage with the insertion of a quite long molecular spacer. The protocol is identical to the one described above for support activation (using proper organic solvent, according to crosslinker solubility:* i.e.*, DMSO or dioxane [35,212]), whereas coupling can be carried out at lower pH values (for instance at neutrality, [213]).

**Scheme 17:**
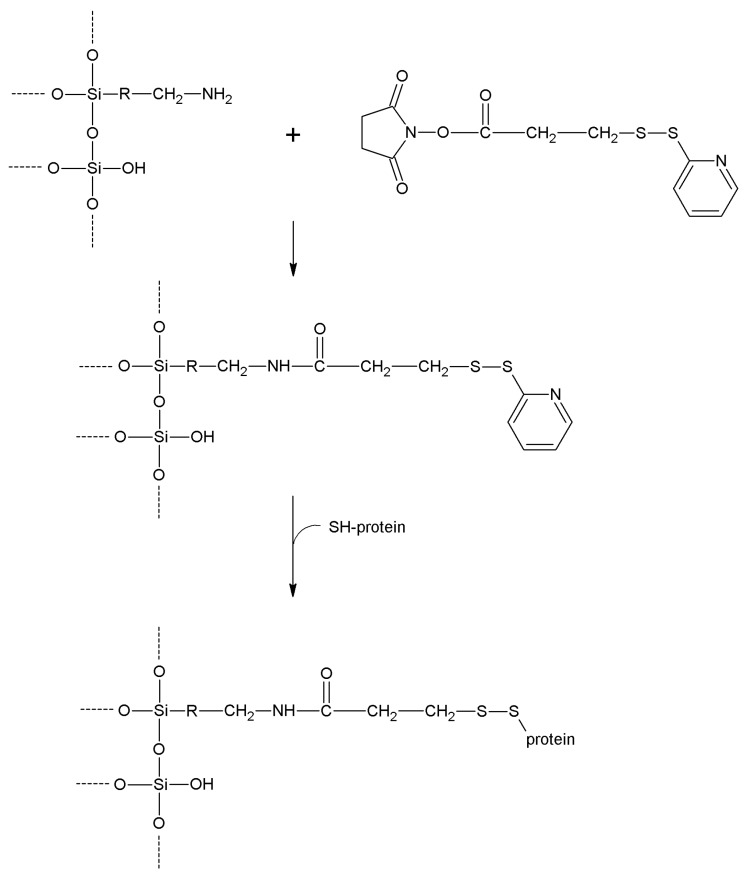
Mechanism of activation using succinimidyl-3-(2-pyridyldithio)propionate (SPDP).

The same pattern of reaction can be accomplished with succinimidyl-3-(2-pyridyldithio)propionate (SPDP), enabling similarly the coupling between alkylamine inorganic supports and protein –SH (Scheme 17). In a slight alkaline aqueous environment (*i.e.*, phosphate buffer pH 8) SPDP previously dissolved in methanol is suspended with a ratio about 1:10 *w/w* with –NH_2_ functionalized support. After 60’ stirring and exhaustive washes, protein coupling occurs about at neutrality [35]. In this case the formed linkage is the less stable disulfide bond. This linkage, on the other side, enables reversible immobilization and easy recovery of the exhausted support.

### 5.8. Activation of Thiol-Functionalized Supports

Thiol-functionalized supports enable specific activation methods, involving coupling with protein –SH (thus, not interfering with lysine amino groups). Since thiol functions are very prone to oxidation during storage, a reducing treatment before any activation is preferable. In a typical protocol, 30’ incubation with 0.03 M dithiothreitol, and 1 mM EDTA is carried out at pH slightly alkaline (*i.e.*, ≈8). Extensive washes are necessary prior to further reaction [35]. EDTA is required to avoid possible oxidation of thiols by heavy metal cation (*i.e.*, iron and copper) contamination.

**Scheme 18:**
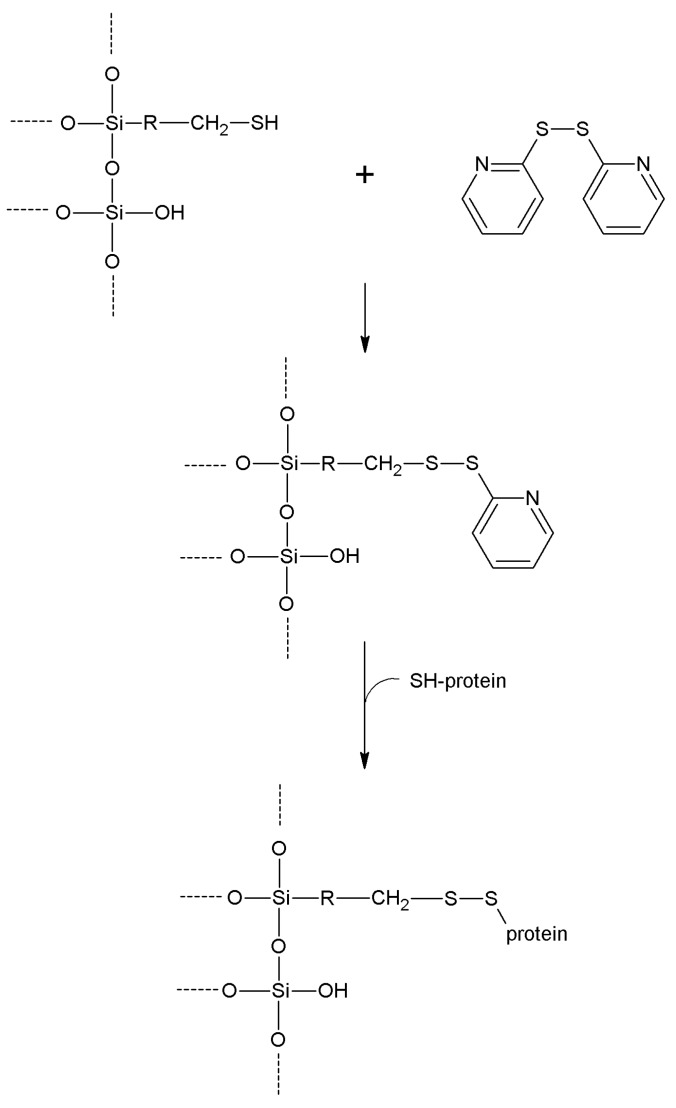
Mechanism of activation using 2-2'-dipyridyldisulfide (DPDS).

2-2'-Dipyridyldisulfide (DPDS) is probably the most used homobifunctional crosslinker for this kind of immobilization procedures. DPDS is very prone to thiol–disulfide exchange because of electron-withdrawing effect by pyridyl nitrogen. A slight alkaline pH (≈8) is necessary to allow the presence of thiolate –S^−^ form of the support [214]. Released 2-thiopyridine is the instable tautomer of 2-thiopyridone, pushing thus the reaction equilibrium towards complete activation of the supports. Activated support in turn couples with protein –SH in another thiol-disulfide exchange reaction (Scheme 18). 

A very short molecular spacer is inserted. The advantage of this approach is the reversibility of the formed bond, enabling the regeneration of costly supports. In a typical protocol, 1.5 mM DPDS is used for support activation at pH 8 in the presence of 1 mM EDTA. After 30’ and exhaustive washes with 1 mM EDTA, coupling with proteins occurs at pH 8 [35].

**Scheme 19:**
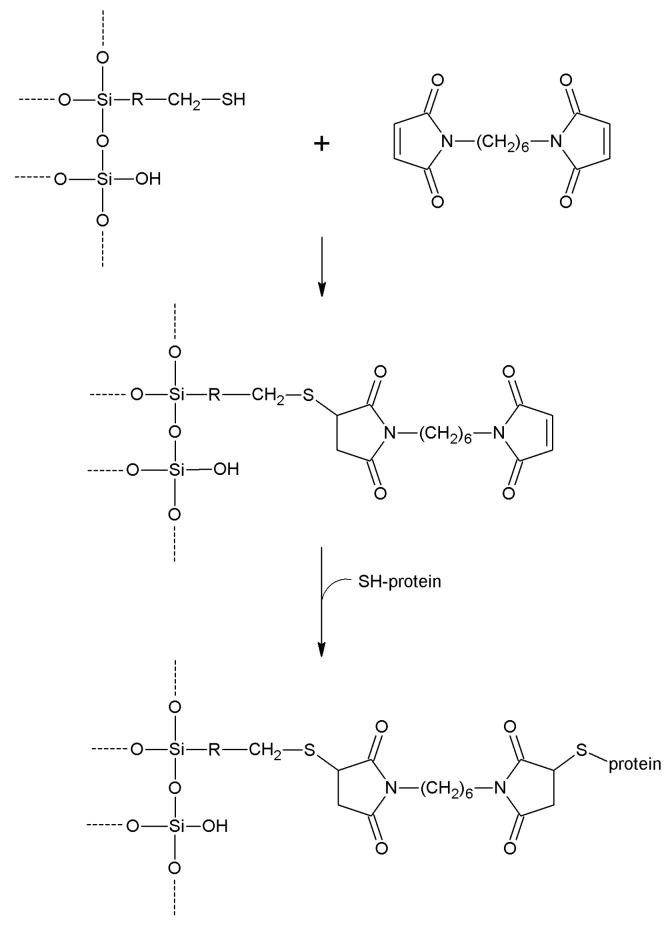
Mechanism of activation using 1,6-bismaleimidohexane (BMH).

Strong and irreversible coupling between carrier and protein –SH can be instead obtained using 1,6-bismaleimidohexane (BMH). The α–β double bond of this crosslinker undergoes nucleophilic addiction in turn by carrier and protein thiols, yielding stable thioether linkages (Scheme 19). In this case, a quite long molecular spacer is also inserted.

BMH requires to be preventively dissolved in a compatible organic solvent (*i.e.*, acetone), and then added to a suspension of the support in a neutral buffer containing 1 mM EDTA [35]. After 60’ stirring and exhaustive washes with EDTA-containing buffer, protein coupling too takes place at neutral pH [35].

### 5.9. Other Activating Methods

Silanol and primary alcoholic –OH groups (*i.e.*, derived from acid hydrolysis of epoxy-functionalized silicas) can be also activated with 1,1'-carbonyldiimidazole (or its analogue 1,1'-carbonyldi-1,2,4-triazole) [215]. This is a quite inexpensive reagent, but very sensitive to hydrolysis. In anhydrous environment (*i.e.*, tetrahydrofuran, acetone or dioxane) excess 1,1'-carbonyldiimidazole reacts with the support for 2 h at room temperature [216]. Crowley and coworkers found that degassing and sonication during this step highly increases the yield of the following coupling [217].

**Scheme 20:**
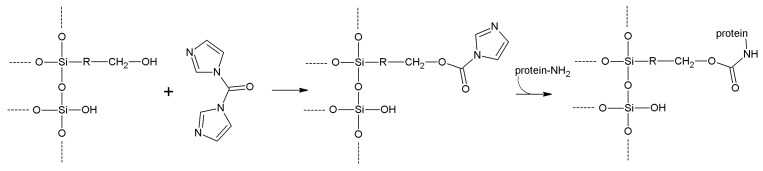
1-1'-Carbonyldiimidazole reacts with –OH from support forming an active ester, able to couple with protein lysines [216].

The activation step yields an activated ester, needing exhaustive washes with organic solvent and then with ice-cold buffer, before protein coupling. Reaction occurs under a wide range of pH values (about 5–10), requiring often several hours [215]. Lysine –NH_2_ groups are usually involved in the final carbamate bond between protein and support (Scheme 20). Such a bond does not present, unfortunately, good stability. The original positive charges of involved lysines are moreover abolished. No any molecular spacer is introduced during activation.

When lysine and/or cysteine are crucial for enzyme native state (and therefore should not be affected by the immobilization procedure), an interesting alternative involves diazotization of the support and coupling with aromatic aminoacid residues. The tyrosine side chain is the most reactive function (coupling takes place at the *ortho* position relative to the phenolic function), but histidine and tryptophan also react, although to a lesser extent [35,218]. The tyrosine reaction is very rapid above its phenolic pKa (10.4) where it exists as a very nucleophilic phenoxide; however, also around neutrality satisfactory reaction rates are observed, being such conditions more compatible with enzyme stability. Azo coupling of tryptophan is often impossible: this is the rarest amino acid and is as a rule buried within the protein core, which renders its coupling quite difficult. 

**Scheme 21:**
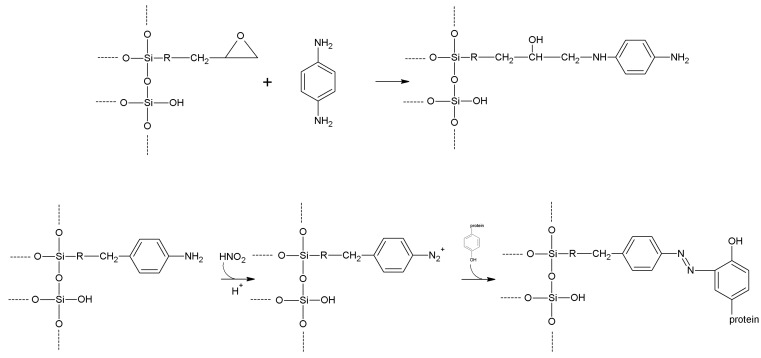
Epoxy-functionalized support can be further functionalized with aromatic amine functions [219], that in turn undergo diazotization and coupling with protein tyrosine phenolic groups [35,218].

The inorganic support needs, however, to be firstly functionalized with aromatic amine functions. This can be accomplished for instance by nitration and reduction of phenyltrialkoxysilane-treated supports (compare § 4.2), or by alkaline reaction between epoxy-activated support and *p*-phenylene-diamine (Scheme 21) [219]. A 1:1 molar ratio between diamine and epoxy functions is advisable. After 2 h at 50 °C support needs exhaustive washes prior to diazotization.

Arylaminated-carriers can be then diazotized in 1% *w/v* sodium nitrite acidified with HCl 1 M [35,218] at 4 °C for at least 30’. After exhaustive washes with cold water, the support is ready for protein coupling at slight alkaline pH [35]. Aromatic carbon from tyrosine acts as a nucleophile towards electrophilic diazonium salt, yielding stable, colored azo bond between protein and support.

A peculiar method of activation involves epichlorohydrin and poly(ethyleneglycol). Since this polymer is commercially available with various degrees of polymerization, it is possible to obtain a molecular spacer with any length, according to the specific desires of operators [220].

**Scheme 22:**
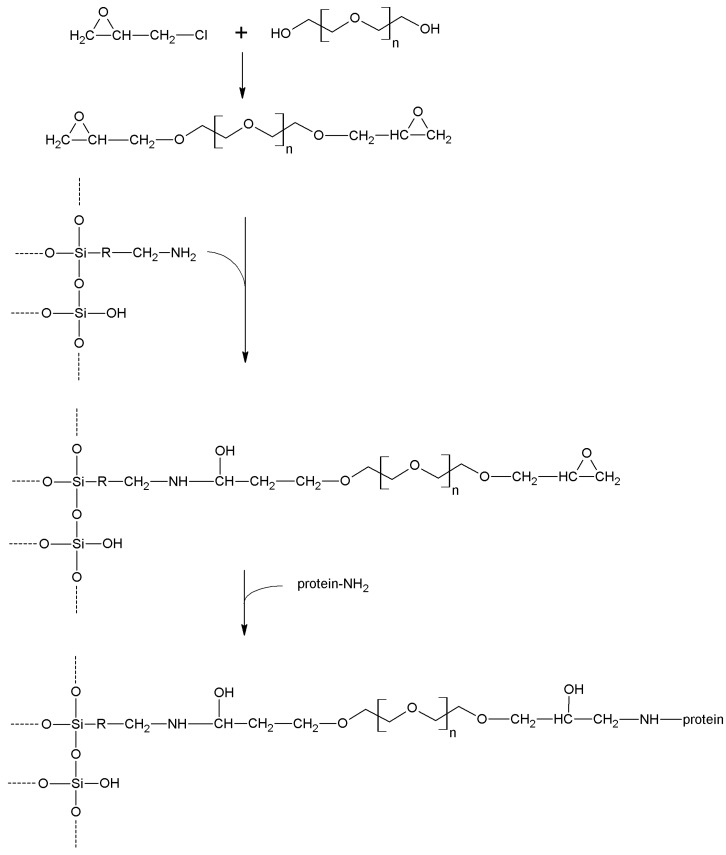
Activation of aminated-silica with epichlorohydrin. Reaction with poly(ethylenglycol) allows the insertion of a molecular spacer long as required [221].

As depicted in Scheme 22, the reaction between epichlorohydrin and poly(ethylenglycol) yields a bifunctional epoxide [221]. This can activate –NH_2_ functionalized supports, that in turn react with protein –NH_2_ groups (*vide infra*, § 5.10), forming a stable secondary amine linkage with a molecular spacer of the desired length.

Epoxy-activated poly(ethylenglycol) is synthesized by aqueous reaction between an excess of epichlorohydrin and a proper length poly(ethylenglycol) for 12 h at 25 °C [221]. The bifunctional reagent is precipitated by solvent evaporation, and used for overnight activation of amino-functionalized support. After exhaustive washes, the epoxy-activated support can be dried and stored at 4 °C, or directly coupled with a protein. The reaction takes place at 25 °C in phosphate buffer pH 7 for 12–24 h [221,222]. Epichlorohydrin can also be used without poly(ethylenglycol) to directly activate –OH functionalized supports, with the insertion of a very small spacer [223].

In a novel technique, Wu and coworkers recently used titania microspheres to covalently immobilize the hemoenzyme catalase through strong coordinative bond among Ti-OH functions and catechol groups [161]. The enzyme was firstly functionalized using the EDC/NHS system (compare § 5.6) and 3-(3,4-dihydroxyphenyl) propionic acid with catechol groups. Then, reaction took place spontaneously at 4 °C and pH 6.5 in 30’ [161]. The approach was quite easy and quick, featured by high loadings and enhanced stability. For instance, Wu and coworkers described that immobilized catalase retained 90% and 76% of its initial activity after 10 and 16 successive catalytic cycles, whereas not significant change of its Michaelis-Menten parameters was observed [160].

### 5.10. Direct Protein Coupling (without Activation)

In some immobilization procedures, however, functionalized supports are able to couple with proteins without any activation. Amino-functionalized supports, for instance, can be used for one-step immobilization without activation or cross-linking agents, using genetically modified enzymes. In their recent paper, Wang and coworkers inserted a genetically encoded CHO–tag into recombinant lipase [224]. Simple incubation for 3 h (phosphate buffer pH 8, room temperature) and reduction of Schiff bases with NaCNBH_3_ led to high immobilization yields and significant stabilization of the enzyme.

The same reaction between amines and aldehydes can be used for –CHO functionalized supports. The so-called glyoxyl functions (in fact, aliphatic aldehyde functions) can be inserted by acidic hydrolysis of epoxy groups: standard protocol requires 2 h treatment with 0.1 M H_2_SO_4_ at 85 °C, exhaustive washings, and 1 h incubation with 0.4 M NaIO_4_ (5 mL per gram of support) at room temperature [225]. Then simple incubation with protein at neutral/mild alkaline pH allows the formation of a Schiff base between protein –NH_2_ and carrier [225,226,227]. Since this bond is quite labile, reduction with sodium borohydride (or its derivatives) is usually recommended, yielding stable secondary amines [225]. Unfortunately, not all proteins are stable under these reducing conditions.

Long coupling times are reported, allowing firstly a physical adsorption. In fact, only physically adsorbed proteins react covalently with –CHO, generating an intense multipoint attachment [226,227]. This approach has been reported to involve minimal leakage and high stabilization [33,225,226]. Only protein –NH_2_ groups can react with glyoxyl functions, so a high density of lysines is required to allow efficient and stable immobilization, which represents the main disadvantage of such an approach. This can be overcome using multimeric proteins (thus, showing several α–NH_2_), or inserting additional amino functions via genetic manipulation or chemical amination [33,169]. An interesting, very particular case is that of lentil seedling amine oxidase being passed onto *N*-aminoethyl-aminopropyl silica. The support is recognized as a substrate by the enzyme, which oxidizes the diamine function thus creating aldehyde groups. These quickly react with the enzyme (which is a glycoprotein) giving rise to irreversible coupling. A satisfactory activity retention is observed [141].

The main reactive moiety able to couple with proteins without activation, however, is the epoxy function. Its reactivity along covalent immobilization and the effect on the tuning of enzyme activity have been recently reviewed [33,228]. Also in this case, low reactivity of the function has been reported, underlining the importance of preventive physical adsorption to obtain intense multipoint attachment and subsequent stabilization [8,228]. Accordingly, quite hydrophobic supports are usually recommended. When this is detrimental for protein activity, blocking of unreacted epoxy functions with hydrophilic molecules can be performed (*i.e.*, mercaptoethanol, ethanolamine, glycine) [33,225,229]. Several protein nucleophile functions are involved in the reaction with carrier epoxy functions: mainly α– and ε–NH_2_, but also –SH, phenol, and imidazole [33,228]. Accordingly, this approach seems to be more versatile than the glyoxyl–based methodology (where only –NH_2_ moieties can be used).

A classical protocol requires suspension of epoxy-activated support in pH 9 buffer for 48 h [35]. An alkaline environment is preferable to enhance the nucleophilic nature of protein reactive groups [33,228], that however need to be present in high number on the protein surface to allow intense multipoint attachment [33].

## 6. Conclusions

Among all immobilization carriers available for researchers operating in the field of enzyme technology, inorganic supports stand out. Some peculiar features, in fact, enable their unmatched application. Microbial resistance, possibly mesoporosity, mechanic and thermal strength are for instance unique characteristics of inorganic supports. In this review, we have encompassed the most widespread supports and the most used approaches for both their functionalization and activation, to give a complete and updated overview of all disposable options. In our opinion, operators should carefully evaluate every feature of both supports and functionalization/activation methods. Only in this way, in fact, covalent immobilization can display all its potential, in the perspective of not random, but instead site-specific procedures aimed to targeted tuning of enzyme properties. With this paper, easy and updated comparison of available resources/procedures is possible, helping operators to make more rational choices.
